# Patterns and Determinants of Prescribing for Parkinson's Disease: A Systematic Literature Review

**DOI:** 10.1155/2019/9237181

**Published:** 2019-11-03

**Authors:** Khalid Orayj, Emma Lane

**Affiliations:** ^1^School of Pharmacy and Pharmaceutical Sciences, Cardiff University, Redwood Building, King Edward VII Ave, Cardiff CF10 3NB, UK; ^2^College of Pharmacy, King Khalid University, Abha, Saudi Arabia

## Abstract

Since the discovery of levodopa (L-dopa) in 1967, the range of medications available to treat Parkinson's disease has increased significantly and guidance on the use, efficacy, and safety of these medications has evolved. To assess levels of adherence to national prescribing guidelines and awareness of changes in the efficacy and safety data published in the profiles of medications for the treatment of PD, we have reviewed studies on patterns and determinants of prescribing PD medications conducted in the last 50 years (since the discovery of L-dopa). A systematic literature review was conducted using EMBASE (1967 to March, 2018), Ovid MEDLINE(R) ALL (1967 to March 16, 2018), PsycINFO (1967 to the 2^nd^ week of March, 2018), and PubMed to identify all studies measuring prescribing patterns of PD medication between 1967 and 2017. Study design, source of data, country, year of study, number of patients and/or prescriptions, unit of analysis, prescribing determinants, and percentage utilisation of PD medications were extracted where possible. 44 studies examining prescribing patterns and/or prescribing determinants across 17 countries were identified. Unsurprisingly, L-dopa was the most commonly prescribed medication in all studies, accounting for 46.50% to 100% of all prescriptions for PD. In several studies, the prescribing rate of ergot-derived dopamine agonists (DAs) decreased over time in concordance with guidance. In contrast, the prescribing rates of non-ergot DAs increased over the last ten years in most of the included studies. In examining prescribing factors, two major categories were exemplified, patients' factors and prescribers' factors, with patients' age being the most common factor that affected the prescription in most studies. In conclusion, L-dopa is now the most commonly prescribed medication for cases of PD but there is large variation in the prescribing rates of catechol-O-methyltransferase (COMT) inhibitors, monoamine oxidase B (MAO-B) inhibitors, amantadine, and anticholinergics between countries. New studies examining the effects of recent clinical trials and measuring the prescribing rates of newly approved medications are warranted.

## 1. Introduction

**Figure 1:**
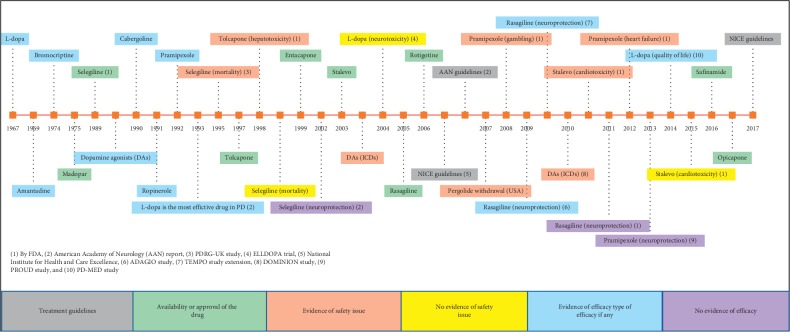
The evolution of pharmacotherapy for Parkinson's disease with key discoveries in efficacy, safety, and approvals of medications since the discovery of L-dopa. The horizontal line represents years from 1967 to 2017. Coloured boxes around the horizontal line represent the event type mentioned in the coloured boxes shown in the bottom of the figure.

Since the first detailed description of the condition now known as “Parkinson's disease” (PD) in 1817, extensive efforts have been devoted to finding a cure. In the late 1960s, George Cotzias described the efficacy and safety of oral levodopa (L-dopa) in treating the motor symptoms of Parkinson's disease. He determined that when the L-dopa dose was increased gradually, motor symptoms improved for a longer duration with minimal gastrointestinal adverse effects [1, 2]. Other compounds were tested alongside L-dopa, including amantadine, which Schwab et al. [3] discovered suppressed tremors. Problematically, although highly effective at treating the motor symptoms, it was determined early on that L-dopa induces dyskinesia and motor fluctuations often develop, limiting use of the drug. There remained a need to search for a drug that could improve motor symptoms without these issues and even more desirable to have disease-modifying properties [4, 5]. In 1974 (see Figure 1), the ergot dopamine agonist, bromocriptine, was tested, demonstrating a longer half-life than L-dopa and fewer motor fluctuations [6]. One year later, a combination of L-dopa and dopa decarboxylase inhibitor (carbidopa) reduced the gastrointestinal side effects compared to L-dopa alone [7–9]. The safety and efficacy of the monoamine oxidase B (MAO-B) inhibitor selegiline (deprenyl), as an adjunct to L-dopa therapy, was then demonstrated in 1977 [10]. From 1982 to 1992, several dopamine agonists (DAs) were introduced to the market, to be used either as L-dopa adjuncts in patients with long-term complications or as *de novo* therapy in place of L-dopa [11]. In 1997, tolcapone, catechol-O-methyl transferase inhibitor (COMT inhibitor) was approved in Europe as a treatment to reduce the motor fluctuations caused by L-dopa [12]. Since then, no new pharmacological class has been introduced in clinical practice; although newer generations of drugs from established drug classes have been introduced, including entacapone (COMT inhibitor) (1999), rasagiline (MAO-B inhibitor) (2005), rotigotine in a patch formulation (non-ergot dopamine agonist) (2006), safinamide (MAO-B inhibitor) (2016), and opicapone (COMT inhibitor) (2016) [12–16]. Additionally, since the early 2000s, new pharmaceutical formulations such as infusion therapies (subcutaneous apomorphine and levodopa-carbidopa intestinal gel (LCIG)) became available in several countries with the promise of tackling the motor complications (mainly the wearing-off phenomenon) caused by the oral form of L-dopa in patients with advanced stage of PD [17].

The perception of the utility of these drugs has evolved over time and this is reflected in subtle changes in the guidance; for example, DAs and MAO-B inhibitors were initially purported to have potential neuroprotective properties leading to their early prescribing following diagnosis but several clinical trials failed to find clear evidence to support this [18–23]. L-DOPA has been widely compared with the DAs, including bromocriptine, ropinirole, pramipexole, and pergolide; these concluded that initiating therapy with DAs was associated with delaying dyskinesia onset or motor fluctuations or both [24–28]. Accordingly, guidelines recommended starting therapy with DAs rather than L-dopa, unless the DAs failed to manage the motor symptoms [29–31] or alternatively commencing therapy with L-dopa or DAs without preference [30, 32]. The impact of the motor fluctuations caused by L-dopa on patients' quality of life (QoL) was not clear until 2014 when the PD-MED study [33] used the quality of life (QoL) scale as a primary outcome. The study's main finding was that early initiation of L-dopa resulted in a better QoL in the long term than initiating DAs and MAO-B inhibitors [33].

Increased knowledge of efficacy and safety and growing number of drugs in the market would be expected to impact prescribing decisions and drug utilisation rates of PD medications. One means through which adherence to national prescribing guidelines and awareness of the changes in efficacy and safety in the medications' profiles can be evaluated by examining prescribing patterns. Doing so would help determine the factors that affect prescribing, including factors such as sex, age, socioeconomic status, education, and drug pricing [34]. Various studies have been conducted worldwide and this review draws together prescribing patterns and determinates of PD medication utilisation across the globe to examine the extent to which these patterns accord with the changes occurring in the safety and efficacy profiles of PD medications.

## 2. Methods

### 2.1. Search Strategy

**Figure 2:**
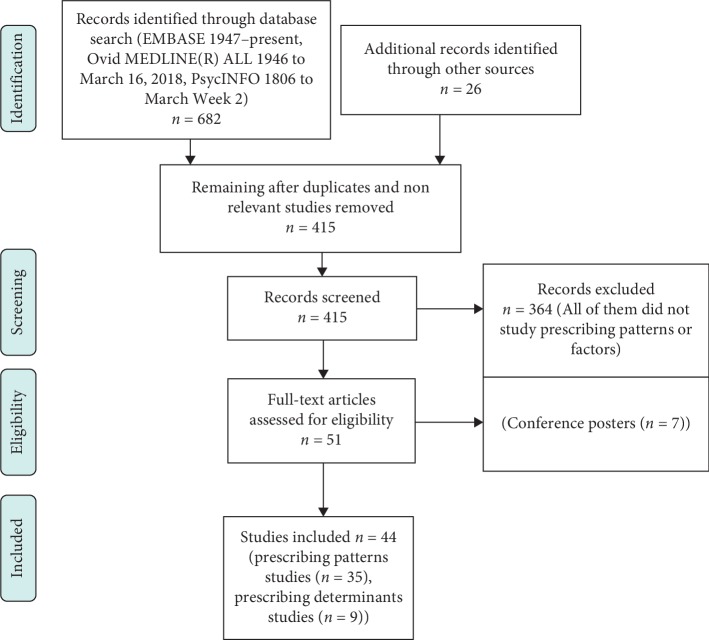
PRISMA flow chart for systematic research of prescribing patterns and determinants study.

A comprehensive and systematic literature search was conducted using EMBASE (1947-March, 2018), Ovid MEDLINE(R) ALL (1946 to March 16, 2018), PsycINFO (1806 to the 2^nd^ week of March, 2018), and PubMed to identify all studies measuring prescribing patterns of PD medications (Figure 2). The key words used were “drug utilization” or “prescribing pattern” or “pharmacoepidemiology” or “prescribing trend” or “inappropriate prescribing” or “prescribing factors” or “prescribing determinants” or “prescribing behaviour,” combined with “Parkinson's disease” or “idiopathic Parkinson's disease” or “Primary Parkinsonism” or “Paralysis Agitans” or “Antiparkinson drugs” or “Antiparkinsonians” or “Antiparkinsonian agents” or “Levodopa” or “L-dopa” or “dopamine agonists” or “apomorphine” or “cabergoline” or “lisuride” or “pergolide” or “pramipexole” or “ropinirole” or “rotigotine” or “amantadine” or “Catechol O-Methyltransferase Inhibitors” or “entacapone” or “tolcapone” or “Monoamine Oxidase Inhibitors” or “rasagiline” or “selegiline” or “anticholinergics or “orphenadrine” or “procyclidine” or “trihexyphenidyl.” Manual reference research and Google Scholar were also used in the review (see Part 1 in Supplementary Materials).

### 2.2. Inclusion and Exclusion Criteria

All English-language studies that measured the prescribing pattern and/or prescribing and drug utilisation determinants of one or more than one class of PD medication at any time point were included in the review. Since the purpose of this review was to examine all prescribing patterns and determinants studies, the only exclusion criterion was if the study was published only as a conference poster. Non-English-language studies were excluded from both the main analysis and the quality assessment due to the lack of translation resources; however, when possible, the English abstracts of these studies were screened and obtained (see Parts 2 and 3 in Supplementary Materials).

### 2.3. Data Extraction, Quality Assessment Checklist, and Data Analysis

Where information was available, the following data were extracted from each study: study design, source of data, country, year of study, number of patients and/or prescriptions, unit of analysis, prescribing determinants, main findings, and utilisation percentages of PD medications. The selected studies were classified into two categories: studies that examined the prescribing patterns of PD medications with or without prescribing determinants and studies that examined prescribing determinants without measuring prescribing patterns of PD medications.

The studies selected for this review had heterogeneous designs which made it difficult to apply the commonly used quality and reporting assessment checklists for cross-sectional observational studies such as the STROPE checklist [35] and the National Institutes of Health Quality Assessment Tool for Observational Cohort and Cross-Sectional Studies [36]. Most published quality and reporting assessment checklists have not been designed to be applied to pharmacoepidemiological and drug utilisation studies [37]. All the studies selected in this review were descriptive in nature and did not measure outcomes caused by exposure in the study participants. For this reason, and to assess and critique the quality of the selected studies, a critical appraisal tool that addresses prevalence studies was used [38]. This tool was chosen as the drug utilisation prevalence of PD medications is the primary interest of this review. The prevalence of PD medication use was used to estimate the prevalence of PD itself in several studies [39, 40]; however, in the current review, it was used solely to study the prescribing patterns and trends of PD medications. For the purposes of this review “The Joanna Briggs Institute Critical Appraisal Tool for Use in Prevalence Studies” was used (see Part 4 in Supplementary Materials). This tool poses 10 questions which can be answered by yes, no, unclear, or not applicable. The questions relate to the sample representativeness of the target population, the study participants recruiting method, the sample size adequacy, the detailed description of study subjects, the sufficiency of the coverage of the selected sample during analysis, the objectivity of the criteria used in measuring the condition, the reliability of the criteria used to measure the condition, the appropriateness of the statistical analysis considering potential confounding factors, and finally, the objectivity of the criteria used to identify subpopulations [38].

After obtaining quality score of each study, the Kruskal–Wallis test was used to compare the prescribing rates at different tiers of quality scores (for this purpose only, quality scores were classified into three tiers: from 1 to 3 and 4 to 6, >6). Additionally, a Kruskal–Wallis test also was used to compare the prescribing rates according to the source of data. The significance level was set at *P* < 0.05 in both tests.

## 3. Results

### 3.1. Search Results and Characteristics of the Drug Utilisation Studies

The initial search of the databases used in this review resulted in the retrieval of 682 studies (see Part 1 in Supplementary Materials). Twenty-six additional studies were identified through other sources (manual reference research and Google Scholar). After removing duplicated and nonrelevant studies, 415 studies remained. The abstracts of these 415 studies were screened and this resulted in the removal of 364 studies which did not examine prescribing patterns or determinants, thus leaving 51 studies. A further 7 studies were excluded as they were published only as conference posters. In total, therefore, 44 studies remained that examined the prescribing pattern and determinants in 17 countries and these were included in this review (Figure 2) [41–84]. Of the 44 studies, 40% (*n* = 18) were undertaken in Europe (Italy (*n* = 4), England (*n* = 2), Germany (*n* = 2), Spain (*n* = 2), Sweden (*n* = 3), Norway (*n* = 2), whole Europe (*n* = 1), Finland (*n* = 1), France (*n* = 1), and UK (*n* = 1)); 29% (*n* = 13) were undertaken in the USA; 25% (*n* = 11) were undertaken in Asia (Japan (*n* = 4), India (*n* = 3), Taiwan (*n* = 2), and China (*n* = 1)), and 7% (*n* = 3) were undertaken in other countries (Australia (*n* = 1), New Zealand (*n* = 1), and South Africa (*n* = 1)). Two studies were conducted in two different countries at once ((USA and Japan jointly) [59] and (Sweden and Norway jointly [84])). This explains why the total of the percentages quoted above exceeds 100% (see Tables 1 and 2). The results of the Kruskal–Wallis tests indicated no significant differences between prescribing rates of PD medications across different levels of study quality scores and across the several data sources that were used in the studies (see Table 3 and Part 5 in Supplementary Materials). The only exception was L-dopa which was prescribed significantly more in studies which used patients' interviews, questionnaires, or surveys compared with studies which used insurance-claims, prescription registries, or drug sales databases (*P* value = 0.011) (Table 3).

Of the 44 studies, 35 were designed to examine the prescribing pattern of PD medications with or without measuring prescribing determinants (Table 1) [41–48, 50, 51, 53–75, 83, 84] and 9 studies measured the prescribing determinants and utilisation factors without measuring prescription rates of PD medications (Table 2) [49, 52, 76–82]. The sources of data varied according to each study design; insurance-claims, prescription registries, or drug sales databases in 16 studies [45–47, 53, 54, 56–58, 61, 62, 64, 65, 72, 75, 81, 83]; medical charts and administrative databases in 13 studies [41–43, 48, 50, 51, 66–70, 79, 82]; patients' interviews, questionnaires, or surveys in 12 studies [44, 55, 59, 60, 63, 71, 73, 74, 76–78, 84]; and finally, 3 studies were designed as post hoc studies that used previously conducted clinical trials to find the prescribing patterns and determinates of PD medications (see Tables 1 and 2) [49, 52, 80]. The timeframe of the studies that were reviewed was from 1986 to 2017. Out of the studies that examined prescribing patterns, 19 were cross sectional in design and calculated the prescription rates of PD medications in a particular period without comparing the rates with other periods [41–45, 48, 50, 55, 57, 60, 64, 68, 69, 71, 73–75, 83, 84] and 15 were designed to compare the prescribing patterns in two or more different periods [46, 47, 51, 53, 54, 56, 58, 59, 61–63, 65–67, 72]. In one study that was conducted in Singapore, the year of the study was not possible to establish [70]. Study settings in prescribing pattern studies varied and included a community setting only (*n* = 20) [42–44, 46, 47, 55, 57, 59, 62, 63, 65–71, 73, 75, 84], inpatient and community settings (*n* = 9) [45, 50, 54, 56, 58, 60, 61, 72, 74], inpatient setting only (*n* = 2) [41, 51], community and care home settings (*n* = 2) [48, 53], inpatient, community, and care home settings (*n* = 1) [64], and finally, care home setting only (*n* = 1) [83]. The general characteristics of the drug prescribing studies that were reviewed are summarised in Table 1. In the prescribing pattern studies, number of patients treated per 100,000 inhabitants, number of prescriptions, number of patients prescribed a particular medication, defined daily doses (DDD) per 1000 inhabitants per day (DID), and number of person-years were used as units of analysis in all studies except one study conducted in England that used drug sales as a unit of analysis [58]. In the studies that used the number of patients prescribed a particular medication [41–43, 45, 47, 48, 51, 54, 55, 59–61, 66, 68–71, 73–75, 83, 84] or the number of person-years [64] as units of analysis, the total prescription rates of all PD medications may not add up to 100% due to the possibility that the patients were prescribed combination therapy. On the contrary, the studies that used the number of prescriptions or DID as units of analysis [53, 57, 62, 65, 67, 72], the total prescription rates of all PD medications may not add up to 100% due to rounding off to the nearest percent or due to the inability to calculate some categories of PD medication prescription rates. One exception to these rules was a study carried out in Taiwan that used the number of prescriptions as a unit of analysis [46]. The total prescription rates of all PD medications exceed 100% due to the fact that some prescriptions include more than one medication. The prescription rates could not be calculated for any of the PD medications in four studies [44, 50, 56, 63].

### 3.2. Quality of the Studies

The quality assessment of the selected studies using the Joanna Briggs Institute Critical Appraisal Tool is illustrated in Part 6 of Supplementary Materials. Out of the prescribing pattern and determinants studies (*n* = 43), two studies were given a quality score of 9 out of 10 (9/10) [45, 49], four studies were given 8/10 [46, 54, 63, 70], seven studies were given 7/10 [51, 56, 68, 73], six studies were given 6/10 [48, 52, 66, 69, 71, 75], eleven studies were given 5/10 [50, 55, 57, 60, 61, 64, 65, 72], ten studies were given 4/10 [42–44, 47, 62, 67, 74], three studies were given 3/10 [41, 53, 58], and finally, one study was given 2/10 [59].

### 3.3. Prescribing Patterns

PD medication prescription rates in all the countries included in this review are presented in Part 7 of Supplementary Materials. Additionally, Table 4 shows a grand summary of PD medications' prescribing pattern.

#### 3.3.1. L-Dopa

All of the studies except five [44, 48, 50, 56, 63] calculated the prescription rate of L-dopa. Out of the studies that calculated L-dopa prescription rates, four calculated the prescription rates of L-dopa-carbidopa and L-dopa-carbidopa-entacapone combinations separately [43, 58, 62, 84]; seven studies calculated the prescription rates of both L-dopa-carbidopa and L-dopa-carbidopa-entacapone combinations altogether without distinction [45, 47, 51, 57, 60, 64, 65], and the rest of the studies calculated only L-dopa-carbidopa prescription rates [42, 46, 53–55, 59, 61, 66–75, 83]. None of the studies that used hospital data mentioned if LCIG prescribing rate was calculated except for one Norwegian study [41]. The Norwegian study found the average number of patients using L-dopa gel to be 2.6 per 100,000 population which was less than the number of patients using deep brain stimulation (DBS) (2.9 per 100,000 population) [41].

Except for a few studies [53, 61, 62, 83], L-dopa was the most commonly prescribed medication in all the studies regardless of the year or the design of the study, accounting for between 37.42% (in Spain) and 100% (in India) of all PD medications [55, 67].

L-dopa prescription rates were the highest (ranging from 46.50% to 100%) compared to other PD medications in several cross-sectional studies in Italy [73, 75], Japan [74], Spain [71], Singapore [70], USA [45, 64, 69], Sweden and Norway [84], South Africa [57], and India [42, 43, 55]. The lowest L-dopa prescription rates were 21% in 2005 and 2008, found in a Japanese study that used the National Japanese Database to examine the effect of pergolide withdrawal from the USA market on PD medication prescribing patterns in Japan by applying a time interrupted series model [61]. L-dopa did not account for the majority of prescription rates in New Zealand (24.86% in 1995) [53] or Australia (36.50% in 1995) [62]. However, both studies reported that L-dopa prescription rates had increased and accounted for the majority of prescribing in 2011 in New Zealand (48.76%) and in 2009 in Australia (52.30%).

**Figure 3:**
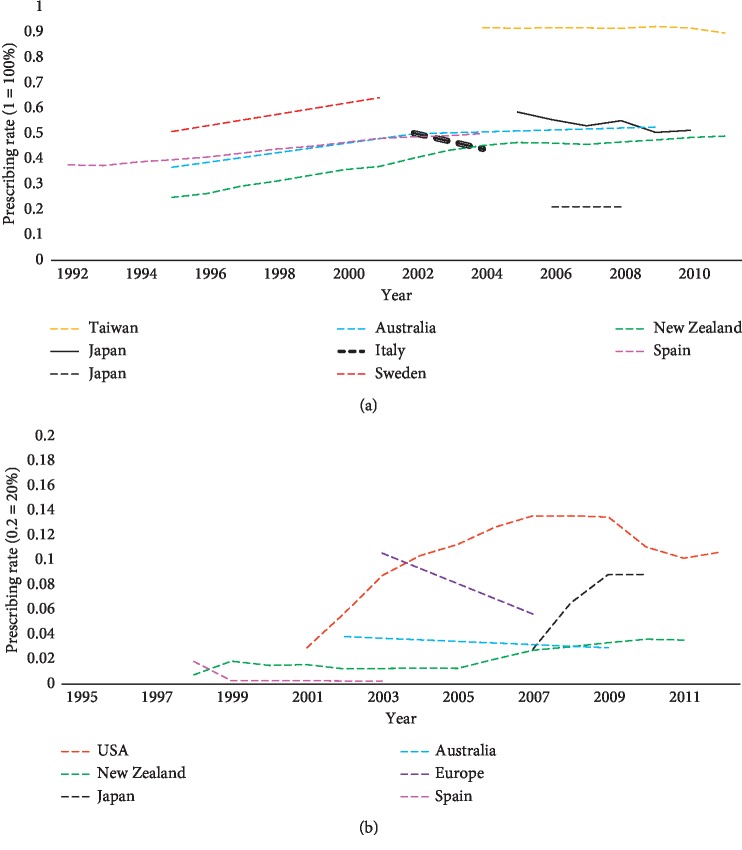
Prescribing trends of PD medications. (a) L-dopa (without entacapone combinations). (b) COMT inhibitors.

Studies carried out in other countries found an increase in the prescription rates of L-dopa in different years.  Figure 3(a) shows that L-dopa prescriptions increased in Sweden, Spain, and Europe in general [65, 67, 72]. Inversely, Figure 3(a) shows a decrease in the prescription rates of L-dopa over the years in Southern Italy, Japan, USA, Finland, and Taiwan [46, 47, 51, 54, 66].

#### 3.3.2. Dopamine Agonists

All bar five studies calculated the prescription rates of DAs (ergot, non-ergot, or both) [44, 50, 56, 63, 75]. Studies that calculated prescribing patterns of DAs can be classified under studies that calculated both ergot and non-ergot DAs prescription rates [43, 51, 53, 54, 58, 61, 62, 66, 67, 71, 73]; DAs prescription rates in general without specifying what type of DAs [42, 45–47, 59, 60, 64, 65, 68, 69, 72, 83, 84]; ergot DAs only [70, 74]; or, non-ergot DAs only [48, 55, 57]. Only four studies examined apomorphine prescribing but without specifying its pharmaceutical formulation forms (subcutaneous injection vs subcutaneous infusion) [53, 62, 67, 73].

**Figure 4:**
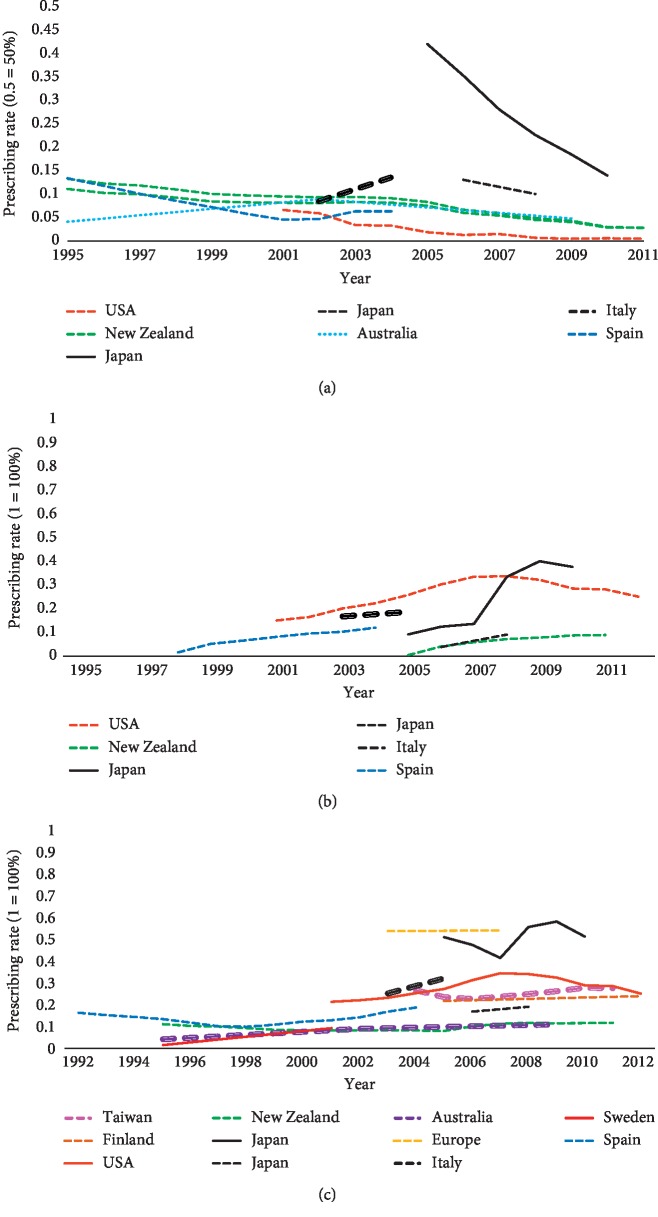
Prescribing trends of PD medications. (a) Ergot dopamine agonists. (b) Non-ergot dopamine agonists. (c) All dopamine agonists.

In general, DAs were the second most common PD medication prescribed after L-dopa in 16 studies with the prescription rate ranging from 7.63% to 85% [45–48, 51, 54, 57, 59, 64, 66, 68–71, 73, 74, 84]. Only one study that examined the pattern of prescribing in nursing homes in five states in the USA found that DAs were the most commonly prescribed PD medication to the members of the study sample, surpassing even L-dopa (75% out of 10,738 PD medications users) [83]. In small number of studies, anticholinergics bumped DAs into third place, ranging from 10.90% to 29% either throughout the study, as in India [42, 43, 55], New Zealand [53], and Japan [61], or at least at one point during the study as in Spain in 1992 [67] and Australia in 2009 [62]. In only 1 retrospective study in Sweden, DAs prescription rates were third after L-dopa and MAO-B inhibitors [72] although DA agonist prescribing continued to grow. Aligned with the Swedish study, a gradual increase in the trend of DAs prescription rates over the years is evident in many countries [46, 47, 51, 53, 61, 62, 66, 67] (see Figure 4(c)). Studies from Australia, New Zealand, Spain, and Italy revealed a slight increase in the use of apomorphine after it became available in these countries [53, 62, 67, 73]. There were no data from other countries regarding apomorphine usage.

*(1) Ergot-Based DAs*. Out of all prescribing pattern studies, thirteen studies calculated the exact prescription rates of ergot DAs [43, 51, 53, 54, 58, 61, 62, 66, 67, 70, 71, 73, 74]. There was a wide range in the prescription rates of ergot DAs which ranged from 0.50% to 76.92%. For studies that calculated the rate of prescribing at only one point of time, there was often an association between the year of the study and the prescription rates. For example, studies carried out prior to 2000 showed higher prescription rates of ergot DAs than these carried out after 2000. Studies that examined the changes in prescription rates across a number of years found a general decrease in prescription rates of ergot DAs [51, 53, 54, 58, 61, 67] ranging from a 3% decrease in prescription rates in Japan between 2005 and 2008 [61] to 30.69% sales costs decrease in England between 1999 and 2010 [58]. The exception was two studies in Australia and Southern Italy show a slight increase in ergot DAs prescription rates [62, 66]. The Australian study revealed an increase in ergot DAs prescription rates from 4.10% in 1995 to 4.80% in 2009 [62] and the Italian study found about 5% increase in the prevalence of ergot DAs use per 100,000 inhabitants between 2003 and 2005 [66] (Figure 4(a)).

*(2) Non-Ergot DAs*. Fourteen studies measured the exact prescription rate of non-ergot DAs [43, 48, 51, 53–55, 57, 58, 61, 62, 66, 67, 71, 73]. Of these, nine calculated the prescription rates at only one time and found that the prescription rates of non-ergot DAs ranged from 5.9% in Australia [62] to 39.80% in South Africa [57]. An increase in the trend of non-ergot DAs prescription rates was observed in several countries [51, 54, 58, 61, 66]. This increase was dramatic in some studies, for instance in England there was a 49.2% increase in non-ergot DAs sales rates between 1999 and 2010 [58]. Typically though, a more modest increase in prescription rates of non-ergot DAs was observed; for instance, in the USA (13% increase between 2001 and 2011) [51], Japan (28.2% increase between 2005 and 2010 or 5.2% increase between 2005 and 2008) [54, 61], and Southern Italy (1.88% increase between 2003 and 2005) [66]. Although there was a general increase in non-ergot DAs prescription rates in an American study carried out in an inpatient setting across a number of years, the prescription rate of non-ergot DAs decreased from 33.4% in 2008 to 27.9% in 2011 following addition of the gambling precaution (which is one of the impulse control disorders (ICDs) forms associated with DAs) to the pramipexole profile in 2008 [51] (Figure 4(b)). Apart from the previous American study (51), only two studies clearly stated the impact of ICDs on non-ergot DAs prescribing and both have shown an increase in non-ergot DAs prescribing in Taiwan (46) and England (58) which revealed no impact of ICDs reports on non-ergots DA prescribing trends.

#### 3.3.3. COMT Inhibitors

The pattern of prescribing of COMT inhibitors was examined in several studies [43, 45, 47, 51, 53–55, 57–62, 64, 65, 67, 69–73]. While only two studies calculated the prescribing rate of the entacapone combination (L-dopa-carbidopa-entacapone combination) with a clear distinction between rates of L-dopa- carbidopa and L-dopa-carbidopa-entacapone combinations [43, 84], several studies have considered L-dopa-carbidopa and L-dopa-carbidopa-entacapone combinations as being one group without clear distinctions [45, 47, 51, 57, 58, 60, 64, 65]. For COMT inhibitor monotherapy, some studies calculated the prescribing rates of tolcapone monotherapy [73], entacapone monotherapy [43, 54, 55, 58, 61, 62, 67, 69, 71], or both [39, 51, 53, 59, 64, 65, 70, 72]. COMT inhibitor monotherapy prescribing in the cross-sectional studies ranged from 1.01% in the USA in 1999-2000 [69] to 29% in the USA in 2003 [59]. An increase was observed in the USA (2.9% in 2001 to 10.6% in 2012) [51], New Zealand (0.73% in 1998 to 3.53% in 2011) [53], and Japan (2.80% in 2007 to 8.80% in 2010) [54]. On the contrary, studies based in Australia, Europe, and Spain showed a slight decrease in COMT inhibitors prescribing [62, 65, 67] (Figure 3(b)). Although a previous study in Europe found a similar decrease in COMT inhibitor prescribing rates, it revealed a significant increase in L-dopa-carbidopa and L-dopa-carbidopa-entacapone combination sales by 68% between 2003 and 2007. As it is accompanied by a decrease in entacapone monotherapy prescribing over the same period, this likely reflects increasing sales of L-dopa-carbidopa-entacapone combinations [65].

#### 3.3.4. MAO-B Inhibitors

**Figure 5:**
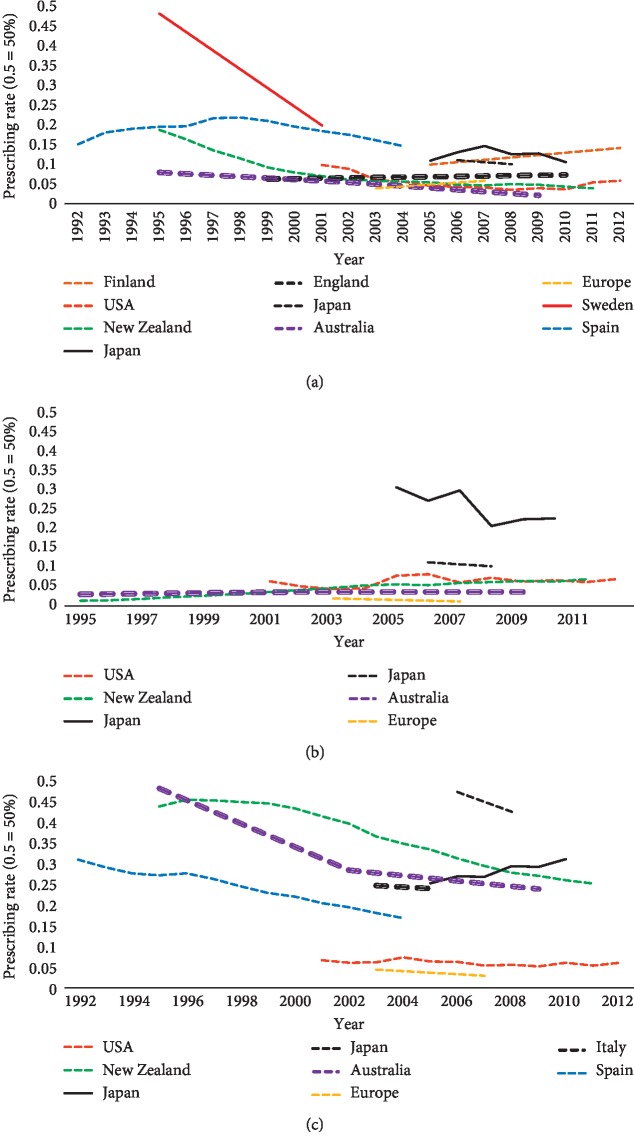
Prescribing trends of PD medications. (a) MAO-B inhibitors. (b) Amantadine. (c) Anticholinergics.

MAO-B inhibitor prescribing patterns were explored in the majority of the identified studies [43, 45, 47, 48, 51, 53–55, 57–62, 64, 65, 67–73, 75, 84]. Out of the two MAO-B inhibitors available, the selegiline prescription rate was measured in 17 studies [53, 54, 57, 60–62, 64, 67–73, 75, 83, 84], both selegiline and rasagiline prescription rates were measured in 6 studies [47, 48, 51, 55, 58, 65], and the rest of the studies measured MAO-B inhibitors as a group without specifying the name of the drug [45, 59]. There were variations in the prescription rates of MAO-B inhibitors in the cross-sectional studies, which ranged from 2.12% in South Africa [57] to 42% in Japan [59]. Other studies that examined changes in the trend of prescription rates over the years revealed varying trends. Selegiline prescribing was either maintained or decreased [53, 54, 61, 62, 67, 72] (Figure 5(a)). Decreases were particularly notable in Sweden between 1995 and 2001 (28% decrease in sales) [72] and New Zealand (18.76% in 1995 to 3.88% in 2011) [53]. A relative steady prescribing rate of selegiline was seen in Japan [54, 61], Australia [62], and Spain [67] (Figure 5(a)). Some studies calculated selegiline rates in the beginning of the study and after calculating both selegiline and rasagiline rates (as a group) when rasagiline became commercially available [47, 51, 58, 65]. Only two studies revealed a slight increase of MAO-B inhibitors prescribing over time (9.90% in 2005 to 14.10% in 2012 in Finland and 3.89% in 2003 to 5.80% in Europe) [47, 65] (Figure 5(a)).

#### 3.3.5. Amantadine

A total of 20 studies measured prescribing rates of amantadine [42, 43, 45, 48, 51, 53–55, 57, 60–62, 64, 65, 68–71, 73, 74]. Among cross-sectional studies, there was wide variation, ranging from 0.2% in Italy [73] to 44.23% in Japan [74]. In trend studies, a steady prescribing rate of amantadine was observed in the USA (6.20% in 2001 and 6.80% in 2012) [51], Australia (2.90% in 1995 and 3.50% in 2009) [62], and Europe (1.86% in 2003 and 1.10% in 2007) [65]. In Japan, 2 studies showed two different trends, i.e., Nakaoka et al. found a decrease in amantadine prescribing from 30% in 2005 to 22.10% in 2010 [54], while Ooba et al. found no major changes between 2006 and 2008 (11% and 10%, respectively) [61]. A noticeable increase in amantadine use was seen in New Zealand (1.26% in 1995 and 6.71% in 2011) [53] (Figure 5(b)).

#### 3.3.6. Anticholinergics

A significant variation was noticed in the cross-sectional studies that examined anticholinergic use in PD patients. Two recent studies in the USA examined anticholinergics prescribing in inpatient and community settings and revealed low prescribing rates of anticholinergics (5% and 6.6%) [45, 64]. This suggests a decreasing trend overall when compared to an earlier study (18.18% between 1992 and 1996) [83]. In some Asian countries (India, Japan, and Singapore), anticholinergics prove more popular with a relatively high prescription rate ranging from 22.9% in Singapore to 40.4% in India [42, 70]. In trend studies, most studies have shown a decrease in prescription rates of anticholinergics across years. This decrease was slight in some countries such as in USA (6.70% in 2001 to 6.10% in 2012) [51]. A more observable decrease was seen in other countries such as in New Zealand (44.30% in 1995 to 25.44% in 2011) [53] (Figure 5(c)).

### 3.4. Prescribing and Drug Utilisation Determinants

Once the determinants of the prescription and utilisation of PD medications are extracted, they could be classified according to patient factors (with several subcategories) and prescriber factors (with only one subcategory). Table 4 shows a summary of prescribing determinants of PD medications.

#### 3.4.1. Patient Factors

*(1) Age*. Several studies have shown that elderly patients (age ≥ 65 years or age ≥ 70 years) were more likely to be prescribed L-dopa than younger patients [45, 47, 51, 54, 57, 60, 63, 69]. The L-dopa dose was inversely associated with age in an examination of 33,534 L-dopa users in Sweden [79]. Moreover, in two studies, elderly patients were less likely to be prescribed [54] or initiated on anticholinergics [68]. In contrast, with the use of L-dopa, and consistent with guidance of preferred L-dopa use in the elderly, the use of DAs was less common in elderly patients [45, 51]. However, there were studies that were discrepant; Crispo et al. found that elderly people in inpatient hospital settings in the USA were regularly prescribed DAs, regardless of national guidelines [51]. Studies have looked at the overall likelihood of receiving PD medications based on age two of which suggested that older patients (>85) were less likely to be medicated [64, 83]. Conversely, Dahodwala et al. similarly in the USA determined that older patients were more likely to receive PD medications than younger patients (OR = 1.67, 95% CI, 1.17–3.27) [81].

On the contrary, a study of younger patients (≤60 years, or ≤ 65 years) revealed a different pattern of prescribing than that pertaining to older patients. Younger patients were more likely to be prescribed DAs in multiple studies and tended to receive more than one medication to treat PD [45, 51, 79]. There is significant country to country variation in the management of younger patients with PD with one US study finding that the majority of younger patients in the study were prescribed L-dopa, while only 20% of younger patients (or ≤ 65 years) were on DAs [68]. MAO-B inhibitors and anticholinergics were more likely to be prescribed as an initial therapy to younger patients than L-dopa in a Taiwanese study [56]. With regard to MAO-B inhibitors, a comparative Italian study that examined 1607 MAO-B users found that rasagiline utilisation was more common in younger patients than selegiline [50]. In trend studies, a Finnish study found that the use of MAO-B inhibitors was increased during the duration of the study (from 2005 to 2011) in younger patients [47].

*(2) Gender*. Multiple studies found no difference between men and women in terms of L-dopa and DAs prescription rates [51, 52, 64, 66, 69]. However, where differences were observed, they were generally indicated men receiving higher doses or to be more likely to receive multiple medications [45, 79, 80, 83]. The effect of gender on the prescribing of other types of PD medications (other than L-dopa and DAs) was not evaluated in all the studies in this review. However, in one study, it was found that rasagiline was more commonly prescribed to men than selegiline, i.e., 45.2% of selegiline users (*n* = 1024) and 57.8% rasagiline users (*n* = 583) were men (*P*=0.001) [50].

*(3) Race*. The effect of patients' race on the prescription and general utilisation of PD medications was only evaluated in the US-based studies. These studies found that, in inpatient and community settings, African American PD patients were less likely to use dopaminergic medications, especially the newer PD medications; prescribed less PD medications; and prescribed more antipsychotics than white Americans [78, 80, 81]. In nursing home settings, African Americans were less likely to receive PD medications in the USA, but this was not statistically significant (OR = 0.89, 95% CI 0.79–1.01) [83]. Another study found that, adding medications that reduce L-dopa-induced motor fluctuations (DAs, MAO-B inhibitors, COMT inhibitors, and amantadine) was more common in non-Hispanic white people when compared with African Americans, although this finding was not statistically significant [82].

*(4) Duration of the Disease*. Some studies measured the duration of the disease as a prescribing determinant. The use of multiple PD medications was positively associated with the duration of the disease in two studies [55, 73]. Another study used data from a clinical trial of creatine versus placebo in participants with early, mild PD (NET-PD LS1) and found that the number of years since PD diagnosis was lower in L-dopa monotherapy users than DAs monotherapy users (1.45 years vs 1.60 years, respectively, *P*=0.02) [49].

*(5) Comorbidities*. Dahodwala et al. found that patients with high morbidity scores (prescription drug hierarchical condition category (RxHCC) risk score) were less likely to receive multiple PD medications (OR = 0.53, 95% CI 0.49–0.57, *P* ≤ 0.001) [45]. Different results were observed in another American study that conducted a logistic regression to find the effect of total comorbidity scores on the chance of receiving single or multiple PD medications in elderly PD Medicare beneficiaries [64]. The study found no association between PD medications use and the total comorbidity score of patients [64]. However, the same study found that some specific types of comorbidities might have an impact on the chance of receiving single or multiple PD medications. For example, patients with depression were more likely to receive PD medications than nondepressed patients (OR = 1.25, 95% CI 1.02–1.53, *P* ≤ 0.05) [64]. On the contrary, patients with dementia were less likely to receive PD medications than nondementia patients (OR = 0.62, 95% CI 0.48–0.80, *P* ≤ 0.001) [64]. Similar findings were observed in nursing home setting in the USA where patients with severe cognitive impairment were less likely to receive PD medications than patients with normal cognitive functions (OR = 0.79, 95% CI 0.73–0.85) [83]. Also, another study found that patients with dementia were prescribed anticholinergics as initial therapy more commonly than non-dementia patients, but this finding was not statistically significant (*P*=0.11) [68]. Another study revealed that the addition of medications that reduce L-dopa-induced motor fluctuations was significantly more common in patients with a high comorbidity score (Charlson Index of 5 or more) (*P*=0.03) [82].

*(6) Socioeconomic Status and Care Settings*. All the studies that examined the effect of socioeconomic status (SES) on PD drug utilisation were conducted in the USA and they reported conflicting results. Yacoubian et al. failed to find an association between PD medication use and educational level, income, and geographical residence of the patients [80]. Another study found no association between PD medication use and income and marital status of the patient [64]. However, the same study revealed that the chance of being prescribed any of the PD medications was higher for patients with a higher education level (high diploma or more) than patients with a lower education level patients (OR = 1.51, 95% CI 1.04–2.19; *P* < 0.05) [64]. Hemming et al. found no difference in the use of PD medications across patients with different levels of income and educational level except for the fact that these with lower income and/or a low education level were less likely to be prescribed newer PD medications and were more likely to be prescribed antipsychotics [78]. Another study found that patients with a higher education level were prescribed DAs more often than patients with a lower education level [49]. With regard to the effect of health insurance on prescriptions, one study carried out in the USA confirmed that PD patients without health insurance received fewer PD medications than patients who had health insurance of any type (*P*=0.0011) [80].

Regarding patients' care settings, an American study found that only 44% of a total of 24,402 nursing home residents with PD in the USA received PD medications [83]. Another US study based on Medicare claims for PD patients from 2000 to 2003 revealed that patients residing in institutions were more likely to receive PD medications than residents within the community (OR = 1.78, 95% CI 1.17–2.71; *P* < 0.01) [64]. The same study found that patients residing in institutions were less commonly prescribed DAs than residents within the community (15.7% vs 35%, respectively) (*P* < 0.001 [64]. In the UK, Hand et al. compared PD medication use in the community vs care homes in a retrospective study using The Northumbria Healthcare NHS Foundation Trust PD service in England [48]. They found that the L-dopa equivalent daily dose (LEDD) prescribed to care home residents was lower (median LEDD = 400 mg, 95% IQR 250–610) than that prescribed to the patients in the community (median LEDD = 657.5 mg, 95% IQR 447.5–1048) (*P* < 0.001) [48]. The same study found that use of DAs, MAO-b inhibitors, and COMT inhibitors was higher in patients living in their homes [48].

*(7) Geographical Location.* This factor has been examined only in one Norwegian study that found that patients who live in Rogaland county were prescribed significantly more L-dopa intestinal gel than other counties in Norway [41]. This difference was attributed in the study to the amount of knowledge patients had about the advanced therapy options in Norway [41].

#### 3.4.2. Prescribers' Factors

*(1) Type of Prescriber*. Eleven studies examined the association between prescriber type and prescribing pattern of PD medications [45, 56, 59, 62, 63, 68, 70–72, 76, 82]. Prescribers in these studies could be classified as general practitioners (GPs), family physicians, mental health providers, geriatricians, neurologists, and movement disorders specialists.

A US survey evaluating 54 family physicians, 328 neurologists, and 74 movement disorder specialists, determined that half of the family physicians and almost one-third of the neurologists prescribe L-dopa as a starting therapy for PD patients immediately after diagnosis [59]. While in Spain, no significant difference was found in the percentages of prescribers of L-dopa among family physicians, geriatricians, neurologists, and movement disorder specialists (87.3%, 86.1%, 91.2%, 91.9%, respectively) [71] although movement disorders specialists tended to prescribe DAs more often and exclusively prescribed amantadine [71]. In USA, family physicians were more likely to prescribe L-dopa, while neurologists and movement disorder specialists were more likely to prescribe DAs [68]. Likewise, in Australia, around 80% of the total DID of L-dopa was prescribed by family physicians while 10% to 20% was prescribed by neurologists with minimal variation between 2003 and 2009 [62].

In the USA, mental health providers were more likely to prescribe anticholinergics as an initial therapy than other prescribers (OR = 76, 95% CI 31.7–181.7) [68], whilst in Spain, the percentage of patients treated with anticholinergics was higher if they were treated by family physicians (17.8%) as opposed to geriatricians (11.1%), neurologists (8.6%), or movement disorder specialists (7%) [71].

Polytherapy and therapy switching were another two issues that only a few studies examined. In USA, Dahodwala et al. found that patients who were treated by neurologists were more likely to receive multiple PD medications than patients who were treated by others (non-neurologists) [45]. In a study in Taiwan that examined the type of initial therapy in PD patients from 2000 to 2010, it was found that 79.3% of L-dopa and DAs combination therapy was initiated by neurologists and 20.7% was initiated by non-neurologists [56]. The same study noted that patients who were treated by neurologists were more likely to be switched to another drug within one year of the study [56].

The impact of the type of prescriber on adherence to national guidelines was another parameter that was evaluated in two studies [63, 76]. The French study failed to find a significant difference between neurologists and non-neurologists in adherence to the type of initial therapy that was recommended in French treatment guidelines of PD in 2000 [63]. Conversely, the Chinese study found that movement disorders specialists were more successful than GPs and general neurologists in improving a patient's quality of care and adhering to Chinese national guidelines that included several recommendations on how to reduce L-dopa-induced motor fluctuations by adding COMT inhibitors, MAO-B inhibitors, or others [76]. Likewise, Cheng et al. found that prescribing medications that reduced L-dopa-induced motor fluctuations was more commonly done by movement disorders specialists than general neurologists and GPs in the USA [82].

## 4. Discussion

The number of PD-related drug utilisation studies identified for review in this study was limited, taking into account the non-negligible prevalence of PD [85, 86]. Most of the studies that were included were conducted in the USA and Europe (68% of all studies) which have limited the geographical spread. This may relate to the high prevalence of PD cases in these countries, exemplified by a recent meta-analysis examining 47 prevalence studies globally, determining that PD prevalence was higher across all ages in Europe, North America, and Australia than in Asia [85]. However, in terms of prevalence, South America surpassed them all [85] but no drug utilisation study in South America was identified for review.

The source of drug utilisation data varied in the reviewed studies, with 38% of the data being sourced from insurance-claim, prescription registry, or drug sales databases. Data sourced from insurance-claims or similar sources may include a large number of patients which makes it possible to generalise the study results to the whole population but it is also highly possible that these databases include patients who have other diseases that have mistakenly been diagnosed as PD (e.g., secondary parkinsonism) since these data lack detailed patient clinical information. Several studies that used this source of data that were included in this review acknowledged this drawback and considered the possibility of over estimation of PD medication prescribing rates [45, 46, 54, 56, 64, 81, 83]. About 26% of the studies reviewed here used patient interviews, questionnaires, and surveys to estimate drug utilisation rates. Although this approach might give a more accurate estimate of medication prescribing patterns, given that the data are based on a more accurate diagnosis by PD experts [43, 44, 63], the relatively small sample sizes restricts the generalisability of the findings. Use of electronic medical records (EMRs) and GP data may overcome the problems of small sample size and misdiagnosis in drug utilisation studies. However, to avoid the inherent drawbacks of EMR (missing data and data entry errors), it is essential to validate these electronic medical records against standard criteria such as the actual paper files of the patients, GP questionnaires, or linking data to other databases [87, 88]. Of all the studies that used EMR and GP data included in this review (28%), none were validated against standard criteria. However, in general, the impact of source of data on PD medications prescribing was minimal in most studies. The exception was L-dopa which was reportedly more prescribed than other PD medications in studies using interviews, questionnaires, and surveys in their methodology. However, this increase is most likely due to the time of these studies (most of them were conducted before 2000), when the current portfolio of dopaminergic drugs was either not clinically available or efficacy was not well-established (Part 5 in Supplementary Materials). Therefore, no valid conclusion could be drawn from a simple comparison of L-dopa prescribing according to the source of data.

In the majority of studies, regardless of the study year or location, unsurprisingly L-dopa plus a dopa decarboxylase inhibitor (carbidopa or benserazide) persists as the most commonly prescribed PD medication, (with or without the COMT inhibitor, entacapone), with no significant changes over time. Where an increase was identified over time (New Zealand, Australia, Sweden, and Spain) [53, 62, 67, 72], this was hypothesised to be due to an increase in PD incidence, an increase in the duration of the disease, or an increasing preference for L-dopa therapy over DAs in the early stages of the disease. Determining which cannot be identified from this data but a real increased PD incidence is unlikely. We had anticipated that some trends might have evidenced the changing recommendations in DA vs L-dopa use. In the early 2000s, multiple studies reported that long-term L-dopa could contribute to neurotoxicity [89, 90]. The ELLDOPA trial in 2004 [91] refuted these findings, and the American Academy of Neurology (AAN) guidelines in 2006 used this evidence to state that L-dopa did not accelerate disease progression [92]. There was vigorous debate in the field at this time on the benefits of commencing therapy with DA agonists to delay onset of L-dopa-induced dyskinesia (LID) and other potential benefits of reduced development of LID we anticipated might have altered prescribing but this was only evident in marginal trends [93, 94]. Recently, the LEAP study revealed that L-dopa has no disease-modifying effects [95]; however, the high reliance on L-dopa could be explained by its higher efficacy and better safety profile than other PD medications [96, 97]. Indeed, the PD-MED study only supported part of this rational used the QoL scale and determining that initiating patients with L-dopa actually resulted in a better QoL than using DAs and that reducing motor fluctuations by delaying L-dopa initiation was not associated with better results over the long term [32].

In this review, there was a noticeable paucity of studies examining the prescribing of advanced infusion therapies in advanced PD patients. For LCIG, there was only one study that examined its prescribing rates in advanced stages of PD [41]. LCIG is an intestinal infusion pump, which aims to avoid the motor complications caused by oral L-dopa, by offering more continuous dopaminergic stimulation and constant levels of plasma levodopa [17]. However, the promising efficacy of LCIG [17] and its improvement to QOL [98] could be hindered by the high cost, invasive nature of the LCIG pump, and possible complications at the infusion site [17]. Therefore, it is of importance to investigate the actual pharmacoepidemiological use of this kind of therapy and the factors that determine its prescribing in future research. It is of importance also to conduct more research on the prescribing of the second type of advanced therapy, apomorphine, since very few studies identified report its prescribing, and those that do fail to specify its pharmaceutical forms.

Early reports of potential neuroprotective effects of DAs may have contributed to a general increase in DAs prescribing in the early 2000s [99–103], but in 2006, the AAN report stated that there was no evidence of neuroprotection for DAs [92] and subsequent reports and clinical trials confirmed the AAN recommendation [17, 20, 22, 103, 104]. These reports might explain why some studies found a slight decrease in the prescription of DAs especially after 2005 [51, 54]. The slight increase or consistent rate in prescribing DAs is seen in other studies [47, 53, 56, 61, 62] which might be due to the fact that DAs were still the recommended treatment in the guidelines as a starting therapy especially with younger patients [105–107]. Recently, the UK NICE guidelines recommended starting therapy with DAs or other dopaminergic therapies (MAO-B inhibitors or L-dopa) in the early stages of PD if the motor symptoms do not impact patients' quality of life [108].

Within the subtypes of the DAs, several cross-sectional studies have shown a wide range of ergot DAs prescription rates. The relatively high prescribing rate seen in the studies conducted before 2000, may be due to the cumulative effect of reports of L-dopa neurotoxicity in the late 1990s and early 2000s [89, 90], the hope that ergot DAs might possess neuroprotective properties [102, 109, 110], and the fact that ergot DAs side effects, such as cardiac fibrosis, had not yet been discovered. In the trend studies, most showed a decrease in the prescription of ergots even though the results of a large-scale UK study that led to a voluntary withdrawal of this drug from the US and Canadian markets in 2007 had not yet been published [111, 112]. For example, in the USA, there was a 5.1% decrease in the prescription of ergots between 2001 and 2007 [51]. The same phenomenon was seen in New Zealand, Japan, Italy, and Spain, in parallel with an increase in non-ergot DAs prescription [53, 54, 66, 71]. An association between the use of ergot DAs (pergolide initially) and valvular heart toxicity was reported in the early 2000s [113]. Whilst non-ergot DAs might have seemed an obvious alternative, reports then emerged of side effects associated with their use [114–118]. Although in several studies the non-ergot prescription rate increased, particularly after pergolide withdrawal [46, 47, 51, 53, 54, 62], the prescription rates decreased in the USA in 2011 [51]. This could be explained by reports of several side effects of non-ergot DAs that appeared between 2006 and 2017. Examples of these side effects, in addition to reports of the risk of heart failure associated with pramipexole, include ICDs [119]. ICDs in this context are typified by a failure to resist the urge for sexual intercourse, gambling, and eating, and it is evident from the DOMINION study that ICDs are significantly associated with DAs [120]. It is disappointing that, despite the increase in recognition ICDs among DAs users, most studies conducted in the last 10 years did not mention the impact of ICDs on DAs prescribing. It will be important to conduct further studies to explicitly examine whether increased awareness of ICDs among prescribers and patients has had an effect on their prescribing.

Prescribing rates of COMT inhibitors were largely consistent with both slight increase [51, 53, 54] and slight decrease [62, 65, 67] reported. In some studies, differentiating the exact prescription rate of COMT inhibitors without considering the L-dopa prescription rate is difficult since the prescription rate of the L-dopa + carbidopa + entacapone combination was reported in the studies but not the rate of entacapone alone [45, 47, 51, 57, 58, 60, 64, 65]. Tolcapone monotherapy was explicitly measured in one study in Italy in 1997-1998 and showed a very low prescription rate (1.3% of the total prescriptions) [73] which is likely linked to the FDA black box warning about the hepatotoxicity risk in 1998 [121] and its very recent approval. Post 2000, any increase in entacapone plus tolcapone prescription rates as in the USA [51] and New Zealand [53] might have been due to entacapone alone since tolcapone prescriptions were restricted due to its hepatotoxicity. No conclusion could be drawn regarding the prescription rates of the L-dopa + carbidopa + entacapone combination in a number of the studies which did not distinguish between its prescription rate and the L-dopa + carbidopa combinations prescription rate [45, 47, 51, 57, 58, 60, 64, 65].

Although it was still in clinical trials testing for possible neuroprotective properties after its approval in 2006, the prescription rate for rasagiline (an MAO-B inhibitor) was only examined in six of the studies [47, 48, 51, 55, 58, 65] whilst the prescription rate for selegiline was highly varied between studies [53, 54, 57, 60–62, 64, 67–73, 75, 83, 84]. The decrease in prescribing around 1995 can be linked to the PDRG-UK trial which suggested association with an increased mortality rate [122], although was subsequently debated by a meta-analysis [123]. Furthermore the decline in use has continued with the purported neuroprotective properties suggested by a range of clinical trials (TEMPO [18], ADAGIO [19]) being unsupported by the guidance [108]. Safinamide is an MAO-B inhibitor that has been recently approved as an add-on therapy to L-dopa in patients who develop motor fluctuations and with its relatively recent appearance its place on the PD stage has yet to evolve significantly.

A huge variation in amantadine prescription rates can be seen, which is characterised by very low and consistent rates in all but Japan for which there is no explanation [54]. Unlike other PD medications, amantadine has not been subjected to significant changes in safety or efficacy profiles since the Schwab trail in 1969 that suggested the clinical efficacy of amantadine in treating PD symptoms [3]. The main indication for amantadine, based on Schwab's work, was to treat the early symptoms of PD, but this was not enough to avoid adding or switching to L-dopa therapy in the long run [124]. In the late 1990s, several studies showed the antidyskinetic effect of amantadine to treat L-dopa-induced dyskinesia [125, 126]. In 2017, the extended release form of amantadine was the first medication that was approved by the US FDA to treat L-dopa-induced dyskinesia [127]. How this formulation and approval affects prescribing remains to be seen moving forward.

Anticholinergics were routinely used in the treatment of PD before the discovery of L-dopa; however, due to their troublesome side effects, their use is limited at present to managing severe tremor in younger patients who do not suffer from cognitive problems [128]. Notwithstanding this fact, anticholinergic prescription rates were generally high in most Asian studies [42, 43, 54, 55, 61, 70] but is generally reducing over time through replacement with other strategies. This was explained, for example, in one Japanese study by the fact that the treatment guidelines in Japan in the early 2000s recommended anticholinergics as the first option [54]. An Indian study attributed this high rate of prescribing anticholinergics to the fact that they were cheaper than most of the other PD medications in India [43]. In the USA, two cross-sectional studies showed a very low rate of anticholinergics prescriptions possibly reflecting an awareness of anticholinergics side effects especially in older patients [45, 64]. Conversely, Lapane et al. found a high rate of anticholinergics prescriptions (18.18%) in nursing home settings in the USA [83]. These data are confounded by the use of anticholinergics in neuroleptic-induced parkinsonism and other conditions.

### 4.1. Prescribing Determinants

Age was one of the most common factors that affects the use of PD medications. In a number of studies, older patients were less likely to receive PD medications than younger patients, likely to be linked to fear of side effects, interactions, or increased morbidity. This is consistent with findings that old age in general has a positive association with high morbidity scores in people with Parkinson's [129].

Whilst L-DOPA has been demonstrated to be the most effective medication for all age groups in PD [130], several studies demonstrated a clear preference for younger patients to be prescribed DA agonists, withdrawing them in older people, consistent with the guidelines. L-dopa causes fewer side effects than DAs in elderly people [131] and DAs are three times more likely to cause hallucinations than L-dopa [132, 133]. Additionally, DAs cause a higher rate of somnolence and sleep attacks [132, 133] and could significantly more likely trigger ICDs such as hypersexuality and pathological gambling [134, 135]. However, notwithstanding these recommendations, Cirspo et al. found that, in inpatient settings in the USA, there was a continuous high rate of prescription of DAs for elderly patients, which raised a question regarding the awareness of treatment guidelines [51]. In relation to the L-dopa dose given, a Swedish study found that older patients were associated with a lower L-dopa dose than younger patients [79] which may be due to the pharmacokinetics (L-dopa has a greater bioavailability and less clearance volume in elderly people [136, 137]).

Overall, according to the studies included in this review, it seems that the several guidelines published after 2000 [28, 107, 138] recommending starting therapy with DAs or MAO-B inhibitors in younger patients and starting L-dopa in older patients might have had an impact on clinical practice. However, according to the results of the PD-MED study, the recent NICE guidelines did not consider age as a factor in choosing the first-line treatment. Patients' quality of life, instead, was the major factor that affected the treatment decision. According to the NICE guidelines, if motor symptoms do not affect patients' quality of life, then starting therapy with DAs or other dopaminergic therapies (MAO-B inhibitors or L-dopa) is recommended [108]. L-dopa, on the contrary, should be used if motor symptoms affect the patients' quality of life [109].

Gender was examined in multiple studies but with conflicting outcomes. Whilst several studies found no gender relationship in L-dopa and DAs prescription rates [51, 52, 64, 66, 69], others found that women had lower odds of being prescribed L-dopa [45], were less likely to receive PD medications (both polytherapy and monotherapy) [45, 80, 83], and the L-dopa daily dose was lower for women [79]. Whilst this may be linked to the pharmacokinetics, this is under research and does require more investigation into the differences in responses between medications and sensitivity to side effects.

In most countries, the patients' race was not investigated as a factor influencing prescription. However, a few studies in USA revealed inequalities relating to African Americans when it comes to PD medication prescriptions, particularly with regard to the newly approved medications, which are generally more expensive [78, 80–83]. Similar inequalities exist across broad tranches of the US healthcare system in relation to PD [139, 140] and other conditions which may be linked to African Americans in general are less likely to have medical insurance and have less access to healthcare facilities than white Americans in the USA [141].

Residence in long-term care facilities such as care homes can be a factor affecting access to health care in PD patients. One study, that included a large number of PD patients in care home settings in the USA, found that about 56% of patients did not receive any PD medication [83]. The study did not consider this phenomenon as a sign of health inequality; rather, it suggested that these patients had most likely been admitted to nursing homes due to debilitating side effects, i.e., psychosis caused by PD medications. This claim was supported by Hand et al. who compared PD medication use in the community vs care homes in England and found that LEDD was lower in care home residents than patients in the community [48]. Although there is a difference in the endpoints of the two previous studies, i.e., the first study examined any single use of PD medication [83] while the second measured the total dose of PD medications taken [48], both reached the same conclusion. According to the two studies, the reason behind the lower use or lower dose of PD medications in care homes was to avoid psychotic episodes caused by PD medication. In PD patients, psychosis can occur as a consequence of the disease itself, or it can be caused by the PD medications [142]. Thus, it is crucial, when managing psychosis in PD patients, to titrate firstly the PD medication doses before considering prescribing antipsychotics [143]. Despite previous evidence that attributed the lack of PD medication utilisation in care homes to a plausible clinical reason, i.e., to avoid the side effects of PD medications, some studies found inappropriate management for PD patients in care homes [83, 144]. This could be explained by lack of access to secondary clinics or switching to a new GP which resulted in suboptimal care [145]. Telemedicine (the approach that uses new technology such as video teleconferencing to link health care providers to PD patients directly) is one tool that could potentially resolve the issue of lacking access to health care due to difficulty accessing health care facilities [146].

Among the countries covered in this review, there were differences in health care systems, prescribing guidelines and in the eligibility of the patients, which limit the value of making comparisons between countries. However, there were some common observations that are worth mentioning in relation to the prescribers themselves. In the only studies identified, movement disorders specialists and neurologists, more than family physicians or GPs, were more likely to prescribe DAs according to some of the studies [62, 68, 71], whereas family physicians and GPs were more likely to prescribe L-dopa or anticholinergics [62, 71]. Since these studies predate many of the changes in guidance, more up-to-date examination of the relative roles and trends in prescribing would be valid.

This study has several limitations. First, the reviewed studies were heterogeneous in terms of design, duration, and data sources. This makes direct comparisons of the prescription rates of different PD medications very difficult. This type of difficulty has been previously identified in other studies [147, 148]. Second, although quality scores were assigned to each study, no study was excluded on the basis of its quality score due to lack of evidence. However, the study score might indicate its quality level. Future studies should focus on developing a quality assessment tool that would help researchers make decisions in drug utilisation research. Third, the fact that this review included only English studies could introduce language bias. However, we tried to minimize this bias by identifying relevant non-English-language studies in our literature searches. The fourth limitation was the assumption that has been made in the discussion section which has attributed the changes in prescribing patterns of PD medication to awareness or nonawareness of the guidelines. Other factors such as drug availability and patient preferences might explain some prescribing behaviours. Therefore, caution should be taken when interpreting the results reported in this review.

## 5. Conclusions

In conclusion, worldwide, since the discovery of L-dopa, it has been the most commonly prescribed PD medication. The prescription rates of ergot-derived DAs decreased in several countries due to cardiac toxicity issues, while the use of non-ergot DAs increased. Significant country to country variation in the prescribing rates of COMT inhibitors, MAO-B inhibitors, amantadine, and anticholinergics was found. Alongside this, patient age was the most common factor that affected prescribing in most studies. The most recent 3^rd^ generation MAO and COMT inhibitors have not been considered in any study as they are so new to the portfolio and new guidance has recently been released in the UK.

## Figures and Tables

**Table 1:** Studies that examined prescribing patterns of PD medications.

Study	Country	Type of study and source of data	PD case ascertainment	Year	Setting	Number of patients and/or prescriptions	Unit of analysis	Prescribing determinants	Comments/main findings	Quality score (out of 10)
Ezat et al. [41]	Norway	Retrospective study from three hospitals in Norway	PD diagnosis confirmed by clinical experts	2009–2013 No comparison	Inpatient setting	262 patients	Number of patients treated per 100,000 inhabitants	Geographical location	Out of all PD medication, the study examined prescribing of L-dopa intestinal gel alone. There is a significant variation of L-dopa intestinal gel prescribing in Norwegian counties (Rosalind county has the highest rate of prescribing).	3

Tripathi et al. [42]	India	Retrospective chart review from a neurology clinic in India	PD diagnosis confirmed by clinical experts	2014 No comparison	Community	100 patients	Percentage of patients prescribed each drug/drug class/drug combinations	N/A	L-dopa monotherapy is the most commonly prescribed regimen. L-dopa + anticholinergic is the second most common regimen followed by L-dopa + DA.	4

Surathi et al. [43]	India	Cross-sectional prescription review study	PD diagnosis confirmed by clinical experts	2011–2014 No comparison	Community	800 patients	Percentage of patients prescribed each drug/drug class/drug combinations	N/A	L-dopa monotherapy is the most commonly prescribed regimen. Anticholinergic is the second most common regimen.	4

Jost et al. [44]	Germany	Cross-sectional surveys with patients and physicians.	PD diagnosis confirmed by clinical experts	2017	Community	4485 patients, and 271 physicians	Percentage of patients prescribed each drug/drug class/drug combinations	N/A	The most commonly prescribed medication is L-dopa (90.27%) followed by DAs (40.66%).	4
Dahodwala et al. [45]	USA	Retrospective cohort from a random sample of annual 5% Medicare Parts A and B claim	Reimbursement data using ICD-9 code. There were efforts to exclude atypical parkinsonism by excluding patients with history of atypical parkinsonism	2007–2010 No comparison	Inpatient and community settings	9482 to 9626 patients	Percentage of patients prescribed each drug/drug class/drug combinations	Age, gender, race, income, comorbidities, and neurology clinic visits.	Most PD patients receive PD medications. African Americans and patients not seen by neurologists are undertreated.	9

Liu et al. [46]	Taiwan	Retrospective cohort from Taiwan National Health Insurance Database	Reimbursement data using ICD-9 code. There were efforts to exclude atypical parkinsonism by excluding patients with history of atypical parkinsonism	2004/2011	Community	19,302 patients in 2004 and 41,606 patients in 2011	Percentage of prescriptions. (one prescription may include more than one prescribed medication)	Age	General increase in L-dopa monotherapy prescribing. More than doubling of DA prescribing for younger patients. Most of the DA prescriptions are non-ergot derivatives after 2008.	8

Keränen and Virta [47]	Finland	Retrospective cohort from a drug insurance reimbursement register	Reimbursement data using ICD-10 code. The reimbursement data were not validated against patient charts.	2005/2012	Community	1436 patients in 2005 and 1607 patients in 2012	Percentage of patients prescribed each drug/drug class	Age	L-dopa is the most prescribed medication in patients aged >75 y. DAs and MOA-B inhibitors are the most prescribed medications in patients aged <60 y. Prescribing changes are in accordance with changes in guidelines.	4
Hand et al. [48]	England	Retrospective study used The Northumbria Healthcare NHS Foundation Trust PD service	PD clinic data. Atypical parkinsonism included, such as multiple system atrophy (MSA) and progressive supranuclear palsy (PSP).	2015	Community and care home settings	377 patients	Percentage of patients prescribed each drug/drug class/drug combinations and L-dopa equivalent daily dose (LEDD)	Care settings	Age and disease stage were higher in those living in care homes. LEDD was lower in those living in care homes. Older age, LEDD, and severe disease stage were significantly associated with care home placement. Use of DAs, MAO-B inhibitors, and COMT inhibitors was higher in patients living in their homes.	6

Degli Esposti et al. [50]	Italy	This retrospective study used databases of three Italian Local Health Authorities	Data linkage study. There were efforts to exclude atypical parkinsonism by linking prescription data to hospital data.	2009–2011 No comparison	Inpatient and community settings	1607 patients on selegiline or rasagiline	Percentage of patients prescribed each drug/drug class/drug combinations	Age and gender	63.3% of patients were on selegiline while 36.2% were on rasagiline. DAs and L-dopa were more prescribed in rasagiline group.	5

Crispo et al. [51]	USA	Retrospective cohort from the Cerner Health Facts database	Hospital diagnosis ICD-9 code. There were efforts to exclude atypical parkinsonism by excluding patients ages less than 40 years	2001/2011	Inpatient	16,785 patients	Percentage of patients prescribed each drug/drug class	Age and gender	L-dopa was the most prescribed medication from 2001 to 2011. Decline in DA use over 2007–2011. Stable rate of DA use in patients aged ≥80 y over 2001–2011.	7
Pitcher et al. [53]	New Zealand	Retrospective cohort from national prescription database in New Zealand	Drug sales database. No efforts to exclude atypical parkinsonism.	1995/2011	Community and rest (care) homes. No comparison.	N/A	Defined daily doses (DDD) per 1000 inhabitants per day	N/A	General increase in L-dopa prescribing over 1995–2011. Slight decrease in DA prescribing over the same interval. Slight increase in COMT inhibitor and amantadine prescribing. An increase in pergolide prescriptions even after 2007.	3

Nakaoka et al. [54]	Japan	Retrospective cohort from medical claim database in JMDC, Tokyo, Japan	Reimbursement data using ICD-10 code. There were efforts to exclude atypical parkinsonism by excluding patients aged <30 years	2005/2010	Inpatient and community settings	714 patients	Percentage of patients prescribed each drug/drug class	Age.	L-dopa is the most prescribed medication over 2005–2010. Of newly diagnosed patients, 30% are prescribed anticholinergics. Non-ergot DA prescribing increases after 2007 in accordance with label revision of ergot DAs.	8

Junjaiah et al. [55]	India	Prospective study that included interviews with PD patients	PD diagnosis confirmed by clinical experts	2011–2013 No comparison	Community	100 patients	Percentage of patients prescribed each drug/drug class/drug combinations	Disease duration	48% of PD patients received L-dopa alone. 52% of PD patients received combination therapies.	5
Guo et al. [56]	Taiwan	Retrospective study used the National Health Insurance Research Database of Taiwan	Reimbursement data using ICD-9 code. There were efforts to exclude atypical parkinsonism by excluding patients who used drugs inducing parkinsonism	2000–2005/2006–2010	Inpatient and community settings	1645 patients	Percentage of patients prescribed each drug/drug class/drug combinations	Age, gender, prescriber type, and interval between PD diagnosis and starting medication	The study examined the initial therapy for newly PD diagnosed patients. L-dopa monotherapy is the most commonly prescribed regimen. DAs were prescribed mainly by neurologists.	7

Gaida et al. [57]	South Africa	Retrospective cohort from national community pharmacy group in South Africa	Drug sales database. There were efforts to exclude atypical parkinsonism by excluding patients aged <50 years	2010 No comparison	Community	5,168 patients and 25,523 prescriptions.	Percentage of prescriptions	Age and gender.	The most commonly prescribed medications are L-dopa + COMT inhibitors. The second most commonly prescribed medications are non-ergot DAs. Patients aged 50–59 y are prescribed DAs more than L-dopa while >70 y group are prescribed more L-dopa.	5

Skogar et al. [84]	Sweden and Norway	Using questionnaires with PD patients and drug registry data	No efforts to exclude atypical parkinsonism.	2010–2013 No comparison	Community	1553 patients in Sweden and 1244 patients in Norway	Percentage of patients prescribed each drug/drug class/drug combinations	NA	L-dopa products were the most commonly used PD medications in both countries. Selegiline was significantly used more in Norway than in Sweden.	4
Morrish [58]	England	Retrospective study that used online statistics at the National Health Service (NHS) Information Centre	Drug sales data. No efforts to exclude atypical parkinsonism	1999/2010	All drug sales in both inpatients and community settings	N/A	Total net ingredient cost for PD medication in pound (£)	N/A	The total net ingredient cost of PD medication was increased from £37 million in 1998 to £130 million in 2010. DAs accounted for the largest portion of overall spending growth. A static spending was seen in L-dopa products. There was a decrease in ergot-DAs spending especially after 2004.	3

Hattor et al. [59]	USA/Japan	Using questionnaires with PD patients followed by interviews with PD patients.	No efforts to exclude atypical parkinsonism.	2003 in USA and 2008 in Japan	Community	300/3548 patients	Percentage of patients prescribed each drug/drug class/drug combinations	Drug side effects.	Patients who had already experienced dyskinesia were less concerned about L-dopa dyskinesia. The most commonly prescribed medication was L-dopa in both countries followed by DAs.	2

Schroder et al. [60]	Germany	A cross-sectional survey of neurologists	PD diagnosis confirmed by clinical experts	2004 No comparison	Inpatient and community settings	60 neurologists complete the medical charts of 320 patients.	Percentage of patients prescribed each drug/drug class/drug combinations	Age and disease severity	53% of patients aged <70 years were used DAs without L-dopa. In patients aged >70 years, 50–52% were used L-dopa without dopamine agonists.	5

Ooba et al. [61]	Japan	Retrospective study used the National Japanese database vendor	Reimbursement data using ICD-10 code. There were efforts to exclude atypical parkinsonism by excluding patients aged <40 years	2005/2008	Inpatient and community settings	547 patients	Percentage of patients prescribed each drug/drug class/drug combinations	Age, gender, and pergolide withdrawal from the USA market in 2007	Percentage of patients prescribed cabergoline or pergolide did not decrease; rather, it tended to increase after 2007.	5
Hollingworth et al. [62]	Australia	Retrospective study using prescription data from Medicare Australia and Drug Utilisation Sub-Committee (DUSC) databases	Reimbursement data. No efforts to exclude atypical parkinsonism	1995/2009	Community	5,078,242 prescriptions	Defined daily doses (DDD) per 1000 inhabitants per day	Age, gender, and type of prescriber	Decline in anticholinergics and DAs over 14 years. General increase in L-dopa use over 14 years. An increase in pramipexole prescribing after 2008.	4

Fayard et al. [63]	France	A population-based study that included interviews with PD patients	PD diagnosis confirmed by clinical experts	≤2000–>2000	Community	308 patients	Percentage of patients prescribed each drug/drug class/drug combinations	Age and type of prescriber.	Agreement with the French recommendations increased after 2000 compared to before 2000. For patients aged <60 years, 35% increase in DAs prescribing after 2000. For patients aged >70 years, about 1% increase in L-dopa prescribing after 2000.	8

Wei et al. [64]	USA	Retrospective study used the Medicare Current Beneficiary Survey and Medicare claims	Reimbursement data using ICD-9 code. The reimbursement data were not validated against patients' charts.	2000–2003 No comparison	Inpatient, community, and nursing home settings	571 patients	Percentage of person-years prescribed each drug/drug class/drug combinations	Age, sex, race, education, marital status, annual income, care setting, and comorbidity scores.	Half of the patients did not use any PD medication in the period of the study. L-dopa was the most PD medication prescribed as a monotherapy or as a combination therapy. Age, prescription drug coverage, residing in an institution, education, dementia, and depression had an effect on PD medication use.	5
Rosa et al. [65]	Europe	Retrospective study that used “intercontinental marketing services” health and examined antiparkinsonian sales in 26 European countries	Drug sales data. No efforts to exclude atypical parkinsonism	2003/2007	Community	A value of 663 million antiparkinsonian consumption in 2003 and 717 million in 2007	Defined daily doses (DDD) per 1000 inhabitants per day	N/A	Levodopa and DAs accounted for half of the drug use in most countries. Between 2003 and 2007, the hugest increase in sales occurred with L-dopa and MAO-B inhibitors. Heterogeneity was seen in the use of PD medications in Europe.	5

Trifiro et al. [66]	Italy	Retrospective study used the Arianna database (GPs database)	GPs and prescription data. GP data were not validated against patients' charts.	2003/2005	Community	1479 patients	Percentage of patients prescribed each drug/drug class/drug combinations	Age	Stable prevalence of PD medication use during the years of the study. L-dopa was the most PD medication prescribed as a monotherapy or as a combination therapy. Non-ergot DAs use was increased in 2005, especially in elderly people.	6

Osinaga et al. [67]	Spain	Retrospective study used the ECOM database of the Spanish Ministry of Health	Drug sales data. No efforts to exclude atypical parkinsonism	1992/2004	Community	N/A	Defined daily doses (DDD) per 1000 inhabitants per day	N/A	L-dopa was the most prescribed PD medication. Consumption of PD medications has increased during the years of the study.	4
Swarztrauber et al. [68]	USA	Retrospective study used the Pacific Northwest Veterans Health Administration (VHA) Data Warehouse	Administrative data using ICD-9 code. The data were validated against patient charts by a neurologist.	1998–2004 No comparison	Community	530 patients	Percentage of patients prescribed each drug/drug class/drug combinations	Age and type of prescriber	29% of the initial antiparkinsonian therapy was initiated by neurologists. 20% of patients younger than 65 years received DAs. Initial antiparkinsonian therapy is strongly influenced by the prescriber's specialty. Additionally, it is mostly initiated by primary care physicians (without PD expertise).	7

Huse et al. [69]	USA	Retrospective study used MedStat's MarketScan Research Databases	Drug registry data using ICD-9 code. No efforts to exclude atypical parkinsonism	1999–2001 No comparison	Community	4846 patients	Percentage of patients prescribed each drug/drug class/drug combinations	Age, gender, comorbidity (Charlson index), and type of insurance.	L-dopa was the most prescribed PD medication as a monotherapy or as a combination therapy regardless of age or type of insurance. DAs are the second most prescribed PD medication, but it only accounted for about 15% of patients younger than 65 years.	6
Tan et al. [70]	Singapore	Retrospective study used patients' charts at a tertiary referral centre. Then factors that influence neurologists' decisions were examined by surveying a sample of neurologists.	PD diagnosis confirmed by clinical experts	N/A	Community	306 patients. 11 neurologists participated in the survey.	Percentage of patients prescribed each drug/drug class/drug combinations	Age, disease severity, intolerance of side effects, drug side effects, drug availability, clinical experience with the drug, drug cost, patient preference, and drug company sponsorship	92.3% of patients were on L-dopa. Most of the patients who were on L-dopa were older and had a higher stage of PD severity scale (Hoen and Yahr). 26.8% of patients were on DAs. From surveying the neurologists, the most important factors influencing their prescribing behaviors were severity of symptoms, intolerance of side effects, and efficacy. The real prescribing behaviours showed a significant positive association of medication usage with cost subsidy by the hospital. There was no mention in the manuscript when this study was conducted, although it was published in 2005.	8

Grandas and Kulisevsky [71]	Spain	A population-based study that included surveying 241 physicians	PD diagnosis confirmed by clinical experts	1999	Community	1803 patients and 241 physicians	Percentage of patients prescribed each drug/drug class/drug combinations	Type of prescriber	L-dopa was the most prescribed PD medication (90.4%) regardless of type of prescriber. DAs were the second common PD medication prescribed (44%). Movement disorder specialists tended to prescribe DAs and COMT inhibitors more than other prescribers followed by neurologists. General physicians used to prescribe anticholinergics more than other prescribers.	6

Askmark et al. [72]	Sweden	Retrospective study that used the prescription sales of 906 community pharmacies and 89 hospital pharmacies.	Drug sales data. PD diagnosis confirmed by clinical experts.	1995/2001	Inpatient and community settings	N/A	Defined daily doses (DDD) per 1000 inhabitants per day	Age and number of neurologists in a particular county	Between 1995 and 2001, L-dopa prescriptions sales increased. After 1997, there has been an increase in sales of DAs (cabergoline, pramipexole, and ropinirole). There was no correlation between the sales of all PD medications and the densities of neurologists or population ages in any particulate county in the study.	5
Leoni et al. [73]	Italy	Cross-sectional surveys with patients.	PD diagnosis confirmed by clinical experts.	1997–1998 No comparison	Community	130 patients	Percentage of patients prescribed each drug/drug class/drug combinations	Age, disease severity, and duration of the disease	L-dopa was the most prescribed PD medication (98.5%) followed by DAs (43.7%). Use of PD medications increased with duration and severity of the disease. Increased age is associated with increased use of PD medications.	7

Lapane et al. [83]	USA	Retrospective study that used (systematic assessment of geriatric drug use via epidemiology) database in 5 states in USA.	Clinical database. Atypical parkinsonism, included such as multiple system atrophy (MSA), and progressive supranuclear palsy (PSP).	1992–1996 No comparison	Nursing homes	24,402 patients	Percentage of patients prescribed each drug/drug class/drug combinations	Gender, race, age and cognitive function	44% of all PD patients in nursing homes received one of the PD medications. DAs were the most common PD medications prescribed (75%) followed by L-dopa (52.27%), MAO-B inhibitor (20.45%), and anticholinergics (18.18). Female, African Americans, and older age patients were less likely to receive PD medication in nursing homes.	7
Fukunaga et al. [74]	Japan	Cross-sectional surveys with patients.	PD diagnosis confirmed by clinical experts.	1994–1996 No comparison	Inpatient and community settings	104 patients	Percentage of patients prescribed each drug/drug class/drug combinations	Duration of the disease	L-dopa was the most prescribed PD medication (78.84%) followed by DAs (76.92%). Combination therapies (2-3 PD medications) were common in patients with duration of disease less than 5 years. The combination therapy of 4 PD medications was common in patients with duration of disease of 7–9 years.	4

Menniti-Ippolito et al. [75]	Italy	Retrospective study that used prescriptions of drugs included in the National Drug Formulary	Atypical parkinsonism, including such as multiple system atrophy (MSA), and progressive supranuclear palsy (PSP).	1986–1991 No comparison	Community	6572 patients	Percentage of patients prescribed each drug/drug class/drug combinations	N/A	L-dopa was the most PD medication prescribed (86.2%) followed by selegiline (24.6%). The main aim of this study was to estimate the prevalence of PD by using the number of patients who used PD medications.	6

**Table 2:** Studies that examined PD medication prescribing determinants only.

Study	Country	Type of study and source of data	PD case ascertainment	Year	Number of patients	Prescribing determinants	Comments/main findings	Quality score (out of 10)
Goudreau et al. [49]	USA	Using data from a clinical trial of creatine vs placebo in participants with early, mild PD on stable doses of dopaminergic therapy (NINDS Exploratory Trials in PD (NET-PD) Long-Term Study-1 (LS1))	PD diagnosis confirmed by clinical experts.	2007–2010 No comparison	1616 patients	Age, gender, race, education level, insurance statue, duration of the disease, comorbidity score, and using of MAO-b inhibitors	This study examined the characteristics of PD patients who enrolled in NET-PD-LS1 study. It compared between patients with L-dopa vs patients with DAs vs patients with a combination therapy (L-dopa + DAs) in terms of proposed prescribing determinates. Higher education level, longer duration of the disease, younger age, and using of MAO-b inhibitors were strongly more common in patients who used DAs.	9

Umeh et al. [52]	USA	Using data from a clinical trial of creatine vs placebo in participants with early, mild PD on stable doses of dopaminergic therapy (NINDS Exploratory Trials in PD (NET-PD) Long-Term Study-1 (LS1))	PD diagnosis confirmed by clinical experts.	2007–2010 No comparison	1741 patients	Gender and education level	This study examined the characteristics of PD patients who enrolled in NET-PD-LS1 study. It compared between patients with L-dopa vs patients with DAs vs patients with a combination therapy (L-dopa + DAs) in terms of proposed prescribing determinates. There was no association between patients' genders and the type of PD medications that were received. There was no association between patients' education levels and the type of PD medications that were received.	6
Chen et al. [76]	China	The cross-sectional questionnaire-based survey was distributed to 612 doctors.	N/A	2010–2011	N/A	Age, type of prescribers, cognitive impairment (CI), and wearing-off phenomenon.	42.9%, 33.5% of doctors preferred using DAs, L-dopa, respectively, for patients aged less than 65 years without CI. 48.3% of doctors preferred switching from immediate release L-dopa to controlled release L-dopa for patient with wearing-off phenomenon. Movement disorder specialists were better than GPs and general neurologists in improving patient quality of care and sticking to national guidelines.	5

Hu et al. [77]	UK	The cross-sectional questionnaire was distributed to 340 PD patients.	PD diagnosis confirmed by clinical experts.	2007–2008	340 patients	Age, cognition, mobility, education level and tremor.	The suboptimal care was defined as (1) more than one year gap between PD diagnosis and first consultation by a specialist and (2) more than one year gap with no evidence of consultant review. Poor cognition, older age, and worse mobility were strongly associated with suboptimal care. Lower educational level and tremor were moderately associated with suboptimal care.	7
Hemming et al. [78]	USA	The cross-sectional questionnaire was distributed to 1090 PD patients	PD diagnosis confirmed by clinical experts.	2003–2008	1090 patients	Race, income, and educational level.	African American PD patients were less likely to use dopaminergic medications and specially the newer PD medications, prescribed less PD medications, and prescribed more antipsychotics compared with white Americans. Generally, there was no difference between using of PD medications across different levels of incomes and educational levels except that these with lower income or/and low educational level were less likely to be prescribed newer PD medications, and they were more likely to be prescribed antipsychotics.	7

Nyholm et al. [79]	Sweden	Retrospective study that used patients' medical files and national drug registries.	PD cases were confirmed by reviewing medical charts	2006–2007	504 patients	Age and gender	The median levodopa daily dose was 465 mg for men and 395 mg for women. The likelihood of dyskinesia was the same in the patients regardless of their total L-dopa dose. Patients' ages were associated inversely with L-dopa dose.	5
Yacoubian et al. [80]	USA	Retrospective study that used the National Institute of Neurological Disorders and Stroke-sponsored REGARDS study.	Using PD medication consumption as a surrogate for PD diagnosis. No efforts to exclude atypical parkinsonism	2003–2007	190 patients	Gender, race, and health insurance	PD patients without health insurance were less likely to receive PD medications. PD medications use was more common in white Americans than African Americans. PD medications use was more common in men compared with women. There was no association between PD medications use and educational level, income, and geographical residence.	4

Dahodwala et al. [81]	USA	Retrospective study that used Pennsylvania State medicaid claims.	Reimbursement data using ICD-9 code. There were efforts to exclude atypical parkinsonism by excluding patients with history of atypical parkinsonism	1999–2003	307 patients	Age, gender, race, county, and type of prescriber.	African Americans were four times less likely to receive PD medications compared with whites. Older age was associated with not receiving PD medications.	4

Cheng et al. [82]	USA	Retrospective study that used an administrative database (the Network 22 VISN Data Warehouse).	Administrative database using ICD-9 code. No efforts to exclude atypical parkinsonism.	2001–2002	309 patients	Age, race, comorbidity (Charlson index), outpatients' visits, and type of prescriber.	An expert panel has determined multiple indicators for quality of PD care including adding DAs, COMT inhibitors, amantadine, and MAO-b inhibitors if the patient developed wearing-off phenomenon. Adherence to previous quality indicator was more common in non-Hispanic white people than African Americans. Adherence to previous quality indicator was associated positively with a high Charlson index, short time from PD diagnosis, more outpatients' visits, and involvement of movement disorder specialists in patient care.	5

**Table 3:** Results of Kruskal–Wallis test for assessing differences in prescribing rates according to the quality score of the studies and source of data.

	L-dopa prescribing rate^a^			COMT inhibitors prescribing rate			All DAs prescribing rate			MAO-B inhibitors prescribing rate			Amantadine prescribing rate			Anticholinergics prescribing rate		
	Median	Range	*P* value^a^	Median	Range	*P* value	Median	Range	*P* value	Median	Range	*P* value	Median	Range	*P* value	Median	Range	*P* value
*Quality score*																		
(1–3)	37.38	26–48.76	0.091	3.58	3.53–3.63	0.245	36.73	11.20–62.26	0.825	5.57	3.88–7.27	0.575	6.71	NA (one study only)	0.895	25.44	NA (one study only)	0.285
(4–6)	70.92	21–100		4.80	0.24–13.31		26.45	9.21–76.92		10.81	2.10–31		5.46	1.10–44.23		5.46	2.91–43	
>6	87.17	51–98.50		6.80	3.10–10.10		28.75	7.63–75		10.50	1.67–21		5.14	0.80–22.10		5.14	3.81–31.40	
*Source of data*																		
Insurance-claims, prescription registries, or drug sales databases	53.78	21–90	**0.009** ^b^	5.61	1–13.31	0.245	29	9.21–75	0.825	9.63	2.10–24.60	0.575	6.58	1.10–22.10	0.895	18.18	2.91–43	0.285
Medical charts and administrative databases	83.60	43.73–94.80		6	0.24–10.10		25	7.63–32.04		8.90	1.67–21		5.10	2–17.20		22.90	3.81–40.40	
Patients' interviews, questionnaires, or surveys	90.40	78.84–100		4	3.10–5.80		43.70	18–76.92		11.08	2.30–31		5	0.80–44.23		19.80	8.50–30.76	

^a^Test based on Kruskal–Wallis statistic, significance level at *P* < 0.05. ^b^Post hoc analysis: insurance-claims, prescription registries, or drug sales databases vs medical charts and administrative databases *P*=0.234. Insurance-claims, prescription registries, or drug sales databases vs patients' interviews, questionnaires, or surveys, *P*=0.011. Medical charts and administrative databases vs patients' interviews, questionnaires, or surveys *P*=0.582.

**Table 4:** Summary of prescribing trends of PD medications and factors associated with their use.

	L-dopa	Dopamine agonists (DAs)	COMT inhibitors	MAO-B inhibitors	Amantadine	Anticholinergics
General prescribing pattern	L-dopa was the most commonly prescribed medication in most studies regardless of the year or the design of the study ranged from 37.42% (in Spain) to 100% (in India). Only one Norwegian study examined the prescribing rate of L-dopa intestinal gel (LCIG).	DAs (non-ergots mainly) were the second most common PD medication prescribed in 16 studies with the prescription rate ranging from 7.63% to 85%. Studies carried out prior to 2000 showed higher prescription rates of ergot DAs than those carried out after 2000. There were no data from most studies regarding apomorphine usage.	Large variation in the prescribing rates of COMT inhibitor monotherapy ranged from 1.01% in USA to 29% in USA as well.	There were variations in the prescription rates of MAO-B inhibitors ranging from 2.12% in South Africa to 42% in Japan.	There was wide variation, ranging from 0.2% in Italy to 44.23% in Japan.	A significant variation was noticed in the cross-sectional studies that examined anticholinergic use in PD. In some Asian countries (India, Japan, and Singapore), anticholinergics prove more popular with a high prescription rate ranging from 22.9% in Singapore to 40.4% in India.

Trend of prescribing across years	There was an increase in L-dopa prescribing across time in Sweden, Spain, and Europe. A decrease in L-dopa prescribing across time was observed in Southern Italy, Japan, USA, Finland, and Taiwan.	A general decrease in prescription rates of ergot DAs and an increase in the trend of non-ergot DAs prescription rates were observed in several countries especially after 2000.	Prescribing increase was observed in the USA, New Zealand, and Japan. On the contrary, studies based in Australia, Europe, and Spain showed a slight decrease in prescribing.	Selegiline prescribing was either maintained or decreased across years. Only two studies revealed a slight increase of MAO-B inhibitors (Rasagline mainly) prescribed over time in Finland and Europe.	Across years, a relatively steady prescribing rate of amantadine was observed in the USA, Australia, and Europe. A general decrease in prescription rates was seen in Japan, and an increase in the trend of prescription rates was observed in New Zealand.	Most studies have shown a decrease in prescription rates of anticholinergics across years

*Patient factors*						
Age	Elderly patients (age ≥ 65 years or age ≥ 70 years) were more likely to be prescribed L-dopa than younger patients.	DAs use was less common in elderly patients with some exceptions as in some USA hospitals.	N/A	Comparative Italian study that examined MAO-B users found that rasagiline utilisation was more common in younger patients than selegiline.	N/A	In two studies, elderly patients were less likely to be prescribed or initiated on anticholinergics.
Gender	Multiple studies found no difference between men and women in the likelihood of L-dopa prescribing.	Multiple studies found no difference between men and women in the likelihood of DAs prescribing.	N/A	One Italian study found that rasagiline was more commonly prescribed to men than selegiline.	N/A	N/A
Race	N/A	In USA, DAs prescribing was more common in non-Hispanic white people when compared to African Americans, although this finding was not statistically significant.	In USA, COMT inhibitors prescribing was more common in non-Hispanic white people when compared to African Americans, although this finding was not statistically significant.	In USA, MAO-B inhibitors prescribing was more common in non-Hispanic white people when compared to African Americans, although this finding was not statistically significant.	In USA, amantadine prescribing was more common in non-Hispanic white people when compared to African Americans, although this finding was not statistically significant.	N/A
Duration of the disease	Number of years since PD diagnosis was lower in L-dopa monotherapy users than DAs monotherapy users.	Number of years since PD diagnosis was lower in L-dopa monotherapy users than DAs monotherapy users.	N/A	N/A	N/A	N/A
Comorbidities	N/A	DAs prescribing was more common in patients with a high comorbidity score.	COMT inhibitor prescribing was more common in patients with a high comorbidity score.	MAO-B inhibitor prescribing was more common in patients with a high comorbidity score.	Amantadine prescribing was more common in patients with a high comorbidity score.	Patients with PD and dementia were prescribed anticholinergics as initial therapy more commonly than non-dementia patients.
Socioeconomic status and care settings	L-dopa equivalent daily dose (LEDD) prescribed to care home residents was lower than that prescribed to the patients in the community.	Patients with a higher education level were prescribed DAs more often than patients with a lower education level. Patients residing in institutions were less commonly prescribed DAs than residents within the community.	COMT inhibitor prescribing was higher in patients living in their homes compared to care homes patients.	MAO-B inhibitor prescribing was higher in patients living in their homes compared to care homes patients.	N/A	N/A
Geographical location	One Norwegian study found that patients living in Rogaland county were significantly prescribed more L-dopa intestinal gel than other counties in Norway.	N/A	N/A	N/A	N/A	N/A
*Prescribers' factors*						
Type of prescriber	In USA, half of the family physicians and almost one third of the neurologists prescribe L-dopa as a starting therapy for PD patients immediately after diagnosis. In Spain, no significant difference was found in the percentages of prescribers of L-dopa among family physicians, geriatricians, neurologists, and movement disorder specialists.	In Spain, movement disorders specialists tended to prescribe DAs more than general practitioners	N/A	N/A	In Spain, movement disorders specialists tended to prescribe amantadine exclusively.	In the USA, mental health providers were more likely to prescribe anticholinergics as an initial therapy than other prescribers. In Spain, the percentage of patients treated with anticholinergics was higher if they were treated by family physicians

## Supplementary Materials

Supplementary Materials Part 1 in the supplementary data shows the search methods for the identification of prescribing pattern studies for antiparkinsonian agents using the following databases (EMBASE, MEDLINE, and PsycINFO). Then, Part 2 shows a PRISMA flow chart for systematic research of prescribing patterns and determinants in non-English-language studies. Next, Part 3 shows a summary of the studies that examined the prescribing patterns of PD medications in non-English-language studies, and Part 4 shows details of the Joanna Briggs Institute Critical Appraisal Tool for Use in Prevalence Studies. Part 5 shows a figure of differences in the prescribing pattern of PD medications according to the quality scores of the studies and data sources. Part 6 shows the quality scores resulting from the Joanna Briggs Institute Critical Appraisal Tool. Finally, Part 7 shows the prescription rates of PD medications in the studies of this review.
