# Supplementary material for: Patterns and Determinants of Prescribing for Parkinson's Disease: A Systematic Literature Review
Source: Parkinsons Dis. 2019 Nov 3;2019:9237181. doi: 10.1155/2019/9237181 (PMC6875178; doi:10.1155/2019/9237181)
Supplement: Supplementary Materials — Part 1 in the supplementary data shows the search methods for the identification of prescribing pattern studies for antiparkinsonian agents using the following databases (EMBASE, MEDLINE, and PsycINFO). Then, Part 2 shows a PRISMA flow chart for systematic research of prescribing patterns and determinants in non-English-language studies. Next, Part 3 shows a summary of the studies that examined the prescribing patterns of PD medications in non-English-language studies, and Part 4 shows details of the Joanna Briggs Institute Critical Appraisal Tool for Use in Prevalence Studies. Part 5 shows a figure of differences in the prescribing pattern of PD medications according to the quality scores of the studies and data sources. Part 6 shows the quality scores resulting from the Joanna Briggs Institute Critical Appraisal Tool. Finally, Part 7 shows the prescription rates of PD medications in the studies of this review. [file 9237181.f1.docx]

**Part 1**

## Search methods for identification of prescribing pattern studies for antiparkinsonian agents (EMBASE, MEDLINE and PsycINFO)

| # | Key word(s) | Results |
| --- | --- | --- |
| 1 | Drug utilization.mp. [mp=ti, ab, hw, tn, ot, dm, mf, dv, kw, fx, nm, kf, px, rx, an, ui, sy, tc, id, tm] | 44235 |
| 2 | Prescribing pattern.mp. [mp=ti, ab, hw, tn, ot, dm, mf, dv, kw, fx, nm, kf, px, rx, an, ui, sy, tc, id, tm] | 1323 |
| 3 | Pharmacoepidemiology.mp. [mp=ti, ab, hw, tn, ot, dm, mf, dv, kw, fx, nm, kf, px, rx, an, ui, sy, tc, id, tm] | 15532 |
| 4 | Prescribing trend.mp. [mp=ti, ab, hw, tn, ot, dm, mf, dv, kw, fx, nm, kf, px, rx, an, ui, sy, tc, id, tm] | 94 |
| 5 | Inappropriate prescribing.mp. [mp=ti, ab, hw, tn, ot, dm, mf, dv, kw, fx, nm, kf, px, rx, an, ui, sy, tc, id, tm] | 6825 |
| 6 | Prescribing factors.mp. [mp=ti, ab, hw, tn, ot, dm, mf, dv, kw, fx, nm, kf, px, rx, an, ui, sy, tc, id, tm] | 30 |
| 7 | Prescribing determinants.mp. [mp=ti, ab, hw, tn, ot, dm, mf, dv, kw, fx, nm, kf, px, rx, an, ui, sy, tc, id, tm] | 5 |
| 8 | Prescribing behavior.mp. [mp=ti, ab, hw, tn, ot, dm, mf, dv, kw, fx, nm, kf, px, rx, an, ui, sy, tc, id, tm] | 1196 |
| 9 | 1 or 2 or 3 or 4 or 5 or 6 or 7 or 8 | 65945 |
| 10 | Parkinson's disease.mp. [mp=ti, ab, hw, tn, ot, dm, mf, dv, kw, fx, nm, kf, px, rx, an, ui, sy, tc, id, tm] | 200952 |
| 11 | Idiopathic Parkinson's disease.mp. [mp=ti, ab, hw, tn, ot, dm, mf, dv, kw, fx, nm, kf, px, rx, an, ui, sy, tc, id, tm] | 7278 |
| 12 | Primary Parkinsonism.mp. [mp=ti, ab, hw, tn, ot, dm, mf, dv, kw, fx, nm, kf, px, rx, an, ui, sy, tc, id, tm] | 44 |
| 13 | Paralysis agitans.mp. [mp=ti, ab, hw, tn, ot, dm, mf, dv, kw, fx, nm, kf, px, rx, an, ui, sy, tc, id, tm] | 1768 |
| 14 | Antiparkinson drugs.mp. [mp=ti, ab, hw, tn, ot, dm, mf, dv, kw, fx, nm, kf, px, rx, an, ui, sy, tc, id, tm] | 470 |
| 15 | Antiparkinsonians.mp. [mp=ti, ab, hw, tn, ot, dm, mf, dv, kw, fx, nm, kf, px, rx, an, ui, sy, tc, id, tm] | 124 |
| 16 | Antiparkinsonian Agents.mp. [mp=ti, ab, hw, tn, ot, dm, mf, dv, kw, fx, nm, kf, px, rx, an, ui, sy, tc, id, tm] | 517 |
| 17 | Levodopa.mp. [mp=ti, ab, hw, tn, ot, dm, mf, dv, kw, fx, nm, kf, px, rx, ui, sy, tc, id, tm] | 74814 |
| 18 | L-Dopa.mp. [mp=ti, ab, hw, tn, ot, dm, mf, dv, kw, fx, nm, kf, px, rx, an, ui, sy, tc, id, tm] | 29406 |
| 19 | Dopamine agonists.mp. [mp=ti, ab, hw, tn, ot, dm, mf, dv, kw, fx, nm, kf, px, rx, an, ui, sy, tc, id, tm] | 20956 |
| 20 | apomorphine.mp. [mp=ti, ab, hw, tn, ot, dm, mf, dv, kw, fx, dq, nm, kf, ox, px, rx, ui, sy, tc, id, tm, mh] | 35382 |
| 21 | Cabergoline.mp. [mp=ti, ab, hw, tn, ot, dm, mf, dv, kw, fx, dq, nm, kf, ox, px, rx, ui, sy, tc, id, tm, mh] | 6943 |
| 22 | lisuride.mp. [mp=ti, ab, hw, tn, ot, dm, mf, dv, kw, fx, dq, nm, kf, ox, px, rx, ui, sy, tc, id, tm, mh] | 4157 |

| # | Key word(s) | Results |
| --- | --- | --- |
| 23 | pergolide.mp. [mp=ti, ab, hw, tn, ot, dm, mf, dv, kw, fx, dq, nm, kf, ox, px, rx, ui, sy, tc, id, tm, mh] | 6318 |
| 24 | pramipexole.mp. [mp=ti, ab, hw, tn, ot, dm, mf, dv, kw, fx, dq, nm, kf, ox, px, rx, ui, sy, tc, id, tm, mh] | 8423 |
| 25 | ropinirole.mp. [mp=ti, ab, hw, tn, ot, dm, mf, dv, kw, fx, dq, nm, kf, ox, px, rx, ui, sy, tc, id, tm, mh] | 5928 |
| 26 | rotigotine.mp. [mp=ti, ab, hw, tn, ot, dm, mf, dv, kw, fx, dq, nm, kf, ox, px, rx, ui, sy, tc, id, tm, mh] | 2736 |
| 27 | Amantadine.mp. [mp=ti, ab, hw, tn, ot, dm, mf, dv, kw, fx, nm, kf, px, rx, an, ui, sy, tc, id, tm] | 21704 |
| 28 | Catechol O-Methyltransferase Inhibitors.mp. [mp=ti, ab, hw, tn, ot, dm, mf, dv, kw, fx, nm, kf, px, rx, an, ui, sy, tc, id, tm] | 1167 |
| 29 | entacapone.mp. [mp=ti, ab, hw, tn, ot, dm, mf, dv, kw, fx, dq, nm, kf, ox, px, rx, ui, sy, tc, id, tm, mh] | 3795 |
| 30 | tolcapone.mp. [mp=ti, ab, hw, tn, ot, dm, mf, dv, kw, fx, dq, nm, kf, ox, px, rx, ui, sy, tc, id, tm, mh] | 2188 |
| 31 | Monoamine Oxidase Inhibitors.mp.[mp=ti, ab, hw, tn, ot, dm, mf, dv, kw, fx, dq, nm, kf, ox, px, rx, ui, sy, tc, id, tm, mh] | 15585 |
| 32 | selegiline.mp. [mp=ti, ab, hw, tn, ot, dm, mf, dv, kw, fx, dq, nm, kf, ox, px, rx, ui, sy, tc, id, tm, mh] | 12833 |
| 33 | rasagiline.mp. [mp=ti, ab, hw, tn, ot, dm, mf, dv, kw, fx, dq, nm, kf, ox, px, rx, ui, sy, tc, id, tm, mh] | 3191 |
| 34 | Anticholinergics.mp. [mp=ti, ab, hw, tn, ot, dm, mf, dv, kw, fx, nm, kf, px, rx, an, ui, sy, tc, id, tm] | 7064 |
| 35 | orphenadrine.mp. [mp=ti, ab, hw, tn, ot, dm, mf, dv, kw, fx, dq, nm, kf, ox, px, rx, ui, sy, tc, id, tm, mh] | 2835 |
| 36 | procyclidine.mp. [mp=ti, ab, hw, tn, ot, dm, mf, dv, kw, fx, dq, nm, kf, ox, px, rx, ui, sy, tc, id, tm, mh] | 1819 |
| 37 | trihexyphenidyl.mp. [mp=ti, ab, hw, tn, ot, dm, mf, dv, kw, fx, dq, nm, kf, ox, px, rx, ui, sy, tc, id, tm, mh] | 7959 |
| 38 | 10 or 11 or 12 or 13 or 14 or 15 or 16 or 17 or 18 or 19 or 20 or 21 or 22 or 23 or 24 or 25 or 26 or 27 or 28 or 29 or 30 or 31 or 32 or 33 or 34 or 35 or 36 or 37 | 344736 |
| 39 | 9 and 38 | 813 |
| 40 | limit 39 to English language | 733 |
| 41 | limit 40 to humans [Limit not valid in PsycINFO; records were retained] | 682 |

##

**Part 2**

PRISMA flow chart for systematic research of prescribing patterns and determinants in non-English studies

Eligibility

(Conference posters excluded [*n* = 3])

Records excluded
*n* = 64 (All of them did not study prescribing patterns or factors)

Identification

Screening

Non-English studies identified in the searches
*n* = 80

Full-text abstract assessed for eligibility
*n* = 7

Studies included *n* = 4

(Chinese language [*n* = 1], German language [*n* = 2], Spanish language [*n* = 1])

Records screened
*n* = 71

Remaining after duplicates *n* = 71 (French language [*n* = 20], German [*n* = 15], Spanish [*n* = 14], Japanese [*n* = 6], Chinese [*n* = 4], ], Dutch [*n* = 4], Finnish [*n* = 2], Italian [*n* = 2], Russian [*n* = 2], Portuguese [*n* = 1], Swedish [*n* = 1])

Included

| **Study** | **Country** | **Type of study** | **Source of data** | **Year** | **Setting** | **Number of patients and/or prescriptions** | **Unit of analysis** | **Prescribing determinants** | **Comments/ Main findings** |
| --- | --- | --- | --- | --- | --- | --- | --- | --- | --- |
| Xiau-hua et al. [1] | China | Retrospective repeated cross sectional | Hospital data (34 hospitals in Nanjing) | 2012/2014  comparison | Hospital | N/A | Defined daily doses (DDD) per 1000 inhabitants per day | N/A | General increase in use of L-dopa and DAs. |
| Fritze [2] | Germany | Retrospective cross sectional | German drug registry. | 2012 | Community | N/A | Defined daily doses (DDD) per 1000 inhabitants per day | N/A | General increase in use of all PD medications  General increase in use of non-ergot DAs. |
| Fritze [3] | Germany | Retrospective cross sectional | German drug registry. | 2011 | Community | N/A | Defined daily doses (DDD) per 1000 inhabitants per day | N/A | The most commonly prescribed medication is L-dopa (90.27%) followed by DAs (40.66%). |
| Montane et al [4] | Spain | Retrospective repeated cross sectional | prescription registry ( ECOM database of the Ministry of Health) | 1989/1998 | Community | N/A | Defined daily doses (DDD) per 1000 inhabitants per day | N/A | General increase in use of selegiline, pergolide, and levodopa. |

**Part 3**

Studies that examined prescribing patterns of PD medications in non-English studies

Reference:

[1] L. Xiao-hua, Z. Z-Young-hong, “Analysis of anti-parkinson disease drugs used in 34 hospitals in Nanjing during 2012—2014,” *Chinese Journal of New Drugs*, vol. 352, no. 12, pp. 1431-1435, 2016.

[2] J.Fritze, “Prescribing patterns of psychotropic drugs in Germany: Results and comments to the Drug Prescription Report 2012,” *Psychopharmakotherapie* , vol. 20, no. 2, pp. 76-81, 2013.

[3] J.Fritze, “Prescribing patterns of psychotropic drugs in Germany: Results and comments to the Drug Prescription Report 2011,” *Psychopharmakotherapie* , vol. 18, no. 6, pp. 245-256, 2011.

[4] E. Montane., A. Ferraz Vallano, and J. M. Castel, "The evolution of use of anti-Parkinson drugs in Spain," *Revista de neurologia* , vol. 34, no. 7, pp. 612-617, 2002.

## **Part 4**

In this review, the sample (patients, prescriptions) would be taken to be representative of the PD population in the area of the study if it covered: (1) patients of all ages; (2) patients of both genders; (3) all disease severity levels; (4) all PD medications available in the area of the study; (5) different morbidities; and (6) different care settings (hospitals, community, and nursing homes). In relation to questionnaires distributed to prescribers, the sample would be considered to be representative if it included at least two types of prescribers (e.g. general practitioners and neurologists) in the study sample. Sample size in this review would be considered adequate if one of the two following conditions were fulfilled: (1) the study used a large national representative sample (national drug claim databases, national electronic medical records, etc.); or, (2) the sample size was calculated in the study. Other than that, the sample size will be considered as not being adequate. In terms of “objectivity of criteria used in measuring the condition”, if the study used an electronic database to obtain the prescribing pattern of PD medications, the database should be validated against standard and accurate databases (medical records, general practitioners (GPs) questionnaire, etc.). If the study used patients’ interviews as a source of information, the diagnosis should be made by an expert in PD medical diagnosis. For other types of methods, the decision is made based on the contents of the article and the measures used to address this issue. With respect to the statistical analyses in the selected studies, if the study examined changes in the trend of PD medications prescription rates, it is expected that an appropriate statistical test that examined the significance of such changes were conducted, e.g. the Cochrane- Armitage test, chi square test, or segmented regression test. If none of these tests were used, the statistical analysis would be deemed inappropriate. On the other hand, if the study examined only the prescribing pattern of PD medications, the descriptive analysis would be deemed appropriate. However, if the study examined the differences between different subgroups in the study, an appropriate statistical test should be applied. With regard to the issue of addressing confounding factors, the study must include at least: age, gender, and disease severity. In relation to all of the criteria mentioned above, the answer “yes” with one score was given if the study fulfilled the criterion; the answers “no”, “unclear”, or “not applicable” with a zero score would be given if the criterion was not fulfilled. After answering all the questions, all the scores were added and a net score was assigned for every study. Due to lack of evidence, no specific quality level (e.g. good, moderate, or poor) was assigned to the selected studies; however, the resulting net score (from 0 to 10) gives an estimation of the quality level of the studies.

**(The following tool was adapted entirely from *Z Munn et al).***

## *The Joanna Briggs Institute Critical Appraisal Tool for Use in Prevalence Studies*

*The 10 criteria used to assess the methodological quality of studies reporting prevalence data and an explanation are described below. These questions can be answered either with a yes, no, unclear, or not applicable.*

*Answers: Yes, No, Unclear or Not/Applicable*

*1. Was the sample representative of the target population?*

*This question relies upon knowledge of the broader characteristics of the population of interest. If the study is of women with breast cancer, knowledge of at least the characteristics, demographics, and medical history is needed. The term “target population” should not be taken to infer every individual from everywhere or with similar disease or exposure characteristics. Instead, give consideration to specific population characteristics in the study, including age range, gender, morbidities, medications, and other potentially influential factors. For example, a sample may not be representative of the target population if a certain group has been used (such as those working for one organisation, or one profession) and the results then inferred to the target population (i.e. working adults).*

*2. Were study participants recruited in an appropriate way?*

*Recruitment is the calling or advertising strategy for gaining interest in the study, and is not the same as sampling. Studies may report random sampling from a population, and the methods section should report how sampling was performed. What source of data were study participants recruited from? Was the sampling frame appropriate? For example, census data is a good example of appropriate recruitment as a good census will identify everybody. Was everybody included who should have been included? Were any groups of persons excluded? Was the whole population of interest surveyed? If not, was random sampling from a defined subset of the population employed? Was stratified random sampling with eligibility criteria used to ensure the sample was representative of the population that the researchers were generalizing to?*

*3. Was the sample size adequate?*

*An adequate sample size is important to ensure good precision of the final estimate. Ideally we are looking for evidence that the authors conducted a sample size calculation to determine an adequate sample size. This will estimate how many subjects are needed to produce a reliable estimate of the measure(s) of interest. For conditions with a low prevalence, a larger sample size is needed. Also consider sample sizes for subgroup (or characteristics) analyses, and whether these are appropriate. Sometimes, the study will be large enough (as in large national surveys) whereby a sample size calculation is not required. In these cases, sample size can be considered adequate.*

*When there is no sample size calculation and it is not a large national survey, the reviewers may consider conducting their own sample size analysis using the following formula:*

*
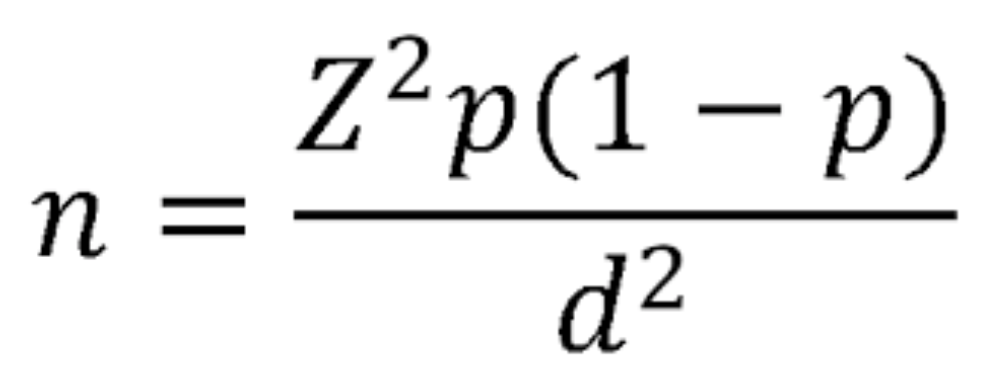
*

*Where:*

*n= sample size*

*Z= Z statistic for a level of confidence*

*P= Expected prevalence or proportion (in proportion of one; if 20%, P= 0.2)*

*d= precision (in proportion of one; if 5%, d= 0.05)*

*4. Were the study subjects and setting described in detail?*

*Certain diseases or conditions vary in prevalence across different geographic regions and populations (e.g. women vs. men, socio-demographic variables between countries). Has the study sample been described in sufficient detail so that other researchers can determine if it is comparable to the population of interest to them?*

*5. Is the data analysis conducted with sufficient coverage of the identified sample?*

*A large number of dropouts, refusals or “not founds” amongst selected subjects may diminish a study’s validity, as can low response rates for survey studies.*

*- Did the authors describe the reasons for non-response and compare persons in the study to those not in the study, particularly with regards to their socio-demographic characteristics?*

*- Could the not-responders have led to an underestimate of prevalence of the disease or condition under investigation?*

*- If reasons for non-response appear to be unrelated to the outcome measured and the characteristics of non-responders are comparable to those in the study, the researchers may be able to justify a more modest response rate.*

*- Did the means of assessment or measurement negatively affect the response rate (measurement should be easily accessible, conveniently timed for participants, acceptable in length, and suitable in content).*

*6. Were objective, standard criteria used for measurement of the condition?*

*Here we are looking for measurement or classification bias. Many health problems are not easily diagnosed or defined and some measures may not be capable of including or excluding appropriate levels or stages of the health problem. If the outcomes were assessed based on existing definitions or diagnostic criteria, then the answer to this question is likely to be yes. If the outcomes were assessed using observer reported, or self-reported scales, the risk of over- or under-reporting is increased, and objectivity is compromised. Importantly, determine if the measurement tools used were validated instruments as this has a significant impact on outcome assessment validity.*

*7. Was the condition measured reliably?*

*Considerable judgment is required to determine the presence of some health outcomes. Having established the objectivity of the outcome measurement instrument (see item 6 of this scale), it is important to establish how the measurement was conducted. Were those involved in collecting data trained or educated in the use of the instrument/s? If there was more than one data collector, were they similar in terms of level of education, clinical or research experience, or level of responsibility in the piece of research being appraised?*

*- Has the researcher justified the methods chosen?*

*- Has the researcher made the methods explicit? (For interview method, how were interviews conducted?)*

*8. Was there appropriate statistical analysis?*

*As with any consideration of statistical analysis, consideration should be given to whether there was a more appropriate alternate statistical method that could have been used. The methods section should be detailed enough for reviewers to identify the analytical technique used and how specific variables were measured. Additionally, it is also important to assess the appropriateness of the analytical strategy in terms of the assumptions associated with the approach as differing methods of analysis are based on differing assumptions about the data and how it will respond. Prevalence rates found in studies only provide estimates of the true prevalence of a problem in the larger population. Since some subgroups are very small, 95% confidence intervals are usually given.*

*9. Are all important confounding factors/ subgroups/differences identified and accounted for?*

*Incidence and prevalence studies often draw or report findings regarding the differences between groups. It is important that authors of these studies identify all important confounding factors, subgroups and differences and account for these.*

*10. Were subpopulations identified using objective criteria?*

*Objective criteria should also be used where possible to identify subgroups (refer to question 6).*

## **Part 5**

Prescribing pattern of PD medications according to quality scores of the studies

## **Part 5 cont**

Prescribing pattern of PD medications according to source of data

**Part 6** Quality appraisal checklist using the Joanna Briggs Institute Critical Appraisal Tool

| **Study^a^** | **A representative sample** | **An appropriate recruitment** | **An adequate sample size** | **Reporting of study subjects and setting** | **Data coverage of the identified sample is adequate** | **Objective, standard criteria used for measurement of the condition** | **The condition was measured reliably and objectively** | **Appropriate statistical analysis** | **Ensuring confounding factors/subgroups/differences are identified and accounted for.** | **Subpopulations identified using objective criteria** | **Quality score** |
| --- | --- | --- | --- | --- | --- | --- | --- | --- | --- | --- | --- |
| Ezat et al. [41] | **UC** | **UC** | **N** | **Y** | **N** | **Y** | **UC** | **Y** | **N** | **N** | **3** |
| Tripathi et al. [42] | **N** | **N** | **N** | **Y** | **NA** | **Y** | **Y** | **Y** | **N** | **N** | **4** |
| Surathi et al. [43] | **N** | **UC** | **UC** | **Y** | **NA** | **Y** | **UC** | **Y** | **Y** | **Y** | **4** |
| Jost et al.[44] | **N** | **N** | **Y** | **Y** | **UC** | **Y** | **UC** | **Y** | **N** | **N** | **4** |
| Dahodwala et al.[45] | **Y** | **Y** | **Y** | **Y** | **Y** | **Y** | **UC** | **Y** | **Y** | **Y** | **9** |
| Liu et al. [46] | **N** | **Y** | **Y** | **Y** | **Y** | **Y** | **UC** | **Y** | **N** | **Y** | **8** |
| Keränen et al. [47] | **N** | **UC** | **Y** | **Y** | **N** | **UC** | **UC** | **Y** | **N** | **Y** | **4** |
| Hand et al. [48] | **N** | **UC** | **Y** | **Y** | **Y** | **N** | **N** | **Y** | **Y** | **Y** | **6** |
| Goudreau et al.[49] | **Y** | **UC** | **Y** | **Y** | **Y** | **Y** | **Y** | **Y** | **Y** | **Y** | **9** |
| Degli Esposti et al. [50] | **N** | **UC** | **Y** | **Y** | **Y** | **UC** | **UC** | **Y** | **N** | **Y** | **5** |
| Crispo et al. [51] | **N** | **Y** | **Y** | **Y** | **Y** | **Y** | **UC** | **Y** | **N** | **Y** | **7** |
| Umeh et al.[52] | **N** | **Y** | **N** | **Y** | **UC** | **Y** | **UC** | **Y** | **Y** | **Y** | **6** |
| Pitcher et al.[53] | **N** | **NA** | **Y** | **Y** | **Y** | **N** | **N** | **N** | **N** | **NA** | **3** |
| Nakaoka et al.[54] | **N** | **Y** | **Y** | **Y** | **Y** | **Y** | **UC** | **Y** | **Y** | **Y** | **8** |
| Junjaiah et al. [55] | **N** | **Y** | **N** | **Y** | **UC** | **Y** | **N** | **Y** | **Y** | **Y** | **5** |
| Guo et al. [56] | **Y** | **UC** | **Y** | **Y** | **Y** | **UC** | **N** | **Y** | **Y** | **Y** | **7** |
| Gaida et al.[57] | **N** | **UC** | **Y** | **Y** | **Y** | **UC** | **N** | **Y** | **N** | **Y** | **5** |
| Skogar et al. [48] | **N** | **UC** | **Y** | **Y** | **Y** | **N** | **N** | **Y** | **N** | **NA** | **4** |
| Morrish [58] | **Y** | **NA** | **Y** | **Y** | **NA** | **N** | **N** | **N** | **N** | **NA** | **3** |
| Hattor et al.[59] | **N** | **UC** | **N** | **Y** | **Y** | **N** | **N** | **N** | **N** | **UC** | **2** |

| **Study^a^** | **A representative sample** | **An appropriate recruitment** | **An adequate sample size** | **Reporting of study subjects and setting** | **Data coverage of the identified sample is adequate** | **Objective, standard criteria used for measurement of the condition** | **The condition was measured reliably and objectively** | **Appropriate statistical analysis** | **Ensuring confounding factors/subgroups/differences are identified and accounted for.** | **Subpopulations identified using objective criteria** | **Quality score** |
| --- | --- | --- | --- | --- | --- | --- | --- | --- | --- | --- | --- |
| Chen et al. [76] | **Y** | **Y** | **UC** | **Y** | **Y** | **NA** | **NA** | **Y** | **N** | **UC** | **5** |
| Schroder et al. [60] | **N** | **Y** | **N** | **Y** | **UC** | **Y** | **UC** | **Y** | **N** | **Y** | **5** |
| Ooba et al. [61] | **N** | **UC** | **Y** | **Y** | **Y** | **N** | **N** | **Y** | **N** | **Y** | **5** |
| Hu et al. [77] | **N** | **Y** | **N** | **Y** | **UC** | **Y** | **Y** | **Y** | **Y** | **Y** | **7** |
| Hollingworth et al. [62] | **N** | **NA** | **Y** | **Y** | **Y** | **N** | **N** | **N** | **N** | **Y** | **4** |
| Hemming et al. [78] | **N** | **N** | **UC** | **Y** | **Y** | **Y** | **Y** | **Y** | **Y** | **Y** | **7** |
| Fayard et al. [63] | **N** | **Y** | **UC** | **Y** | **Y** | **Y** | **Y** | **Y** | **Y** | **Y** | **8** |
| Wei et al.[64] | **N** | **UC** | **Y** | **Y** | **Y** | **N** | **N** | **Y** | **N** | **Y** | **5** |
| Rosa et al. [65] | **Y** | **NA** | **Y** | **Y** | **Y** | **N** | **N** | **Y** | **N** | **NA** | **5** |
| Nyholm et al. [79] | **Y** | **NA** | **Y** | **Y** | **UC** | **N** | **N** | **Y** | **N** | **Y** | **5** |
| Yacoubian et al. [80] | **N** | **UC** | **UC** | **Y** | **Y** | **N** | **N** | **Y** | **N** | **Y** | **4** |
| Dahodwala et al. [81] | **N** | **UC** | **N** | **Y** | **UC** | **Y** | **N** | **Y** | **N** | **Y** | **4** |
| Trifiro et al. [66] | **N** | **UC** | **N** | **Y** | **Y** | **Y** | **Y** | **Y** | **N** | **Y** | **6** |
| Cheng et al. [82] | **N** | **UC** | **N** | **Y** | **Y** | **N** | **N** | **Y** | **Y** | **Y** | **5** |
| Osinaga et al. [67] | **Y** | **NA** | **Y** | **Y** | **Y** | **N** | **N** | **N** | **N** | **NA** | **4** |
| Swarztrauber et al.[68] | **N** | **UC** | **Y** | **Y** | **Y** | **Y** | **Y** | **Y** | **N** | **Y** | **7** |
| Huse et al. [69] | **N** | **UC** | **Y** | **Y** | **Y** | **N** | **N** | **Y** | **Y** | **Y** | **6** |
| Tan et al. [70] | **N** | **Y** | **N** | **Y** | **Y** | **Y** | **Y** | **Y** | **Y** | **Y** | **8** |
| Grandas et al. [71] | **Y** | **UC** | **Y** | **Y** | **Y** | **UC** | **UC** | **Y** | **N** | **Y** | **6** |

| **Study^a^** | **A representative sample** | **An appropriate recruitment** | **An adequate sample size** | **Reporting of study subjects and setting** | **Data coverage of the identified sample is adequate** | **Objective, standard criteria used for measurement of the condition** | **The condition was measured reliably and objectively** | **Appropriate statistical analysis** | **Ensuring confounding factors/subgroups/differences are identified and accounted for.** | **Subpopulations identified using objective criteria** | **Quality score** |
| --- | --- | --- | --- | --- | --- | --- | --- | --- | --- | --- | --- |
| Askmark et al. [72] | **Y** | **NA** | **Y** | **Y** | **Y** | **UC** | **N** | **N** | **N** | **Y** | **5** |
| Leoni et al. [73] | **N** | **UC** | **N** | **Y** | **Y** | **Y** | **Y** | **Y** | **Y** | **Y** | **7** |
| Lapane et al. [83] | **N** | **Y** | **Y** | **Y** | **Y** | **Y** | **UC** | **Y** | **N** | **Y** | **7** |
| Fukunaga et al. [74] | **N** | **UC** | **N** | **Y** | **UC** | **Y** | **N** | **N** | **Y** | **Y** | **4** |
| Menniti-Ippolito et al.[75] | **Y** | **Y** | **N** | **Y** | **Y** | **N** | **N** | **NA** | **Y** | **Y** | **6** |

1. Yes (Y), No (N), Unclear (UC) or Not/Applicable (NA)

Part 7- PD medications prescription rates*

| **Country** | **Year** | **L-dopa^d^ only^a^** | **L-dopa combination^b^** | **COMT inhibitors** | **Ergot DAs** | **Non-ergot DAs** | **All DAs** | **MAO-B inhibitors** | **Amantadine** | **Anticholinergics** |
| --- | --- | --- | --- | --- | --- | --- | --- | --- | --- | --- |
| Norway^e^ [41] | 2009-2013 | ⎯^c^ | ⎯ | ⎯ | ⎯ | ⎯ | ⎯ | ⎯ | ⎯ | ⎯ |
| India [42] | 2014 | 94.8 | ⎯ | ⎯ | ⎯ | ⎯ | 23.2 | ⎯ | 17.2 | 40.4 |
| India [43] | 2011-2014 | 86 | 92 | 6 | 2 | 27 | 29 | 12 | 2 | 31 |
| Germany ^f^ [44] | 2017 | ⎯ | ⎯ | ⎯ | ⎯ | ⎯ | ⎯ | ⎯ | ⎯ | ⎯ |
| USA ^g^ [45] | 2010 | ⎯ | 90 | 6 | ⎯ | ⎯ | 29 | 11 | 7 | 5 |
| Taiwan [46] | 2004/2011 | 91.38/89.24 | ⎯ | ⎯ | ⎯ | ⎯ | 26.52/27.54 | ⎯ | ⎯ | ⎯ |
| Finland [47] | 2005/2012 | ⎯ | 59/55.30 | ⎯ | ⎯ | ⎯ | 21.70/23.9 | 9.90/14.10 | ⎯ | ⎯ |
| England [48] | 2015 | ⎯ | ⎯ | ⎯ | ⎯ | 38.5/13.2^h^ | 38.5/13.2 | 12.2/1.1 | 7.3/4.4 | ⎯ |
| Italy^i^ [50] | 2009-2011 | ⎯ | ⎯ | ⎯ | ⎯ | ⎯ | ⎯ | ⎯ | ⎯ | ⎯ |
| USA [51] | 2001/2011 | ⎯ | 89.10/85.10 | 2.90/10.10 | 6.60/0.50 | 14.90/27.90 | 21.5/28.50 | 9.80/5.80 | 6.20/6 | 6.70/5.40 |
| New Zealand [53] | 1995/2011 | 24.68/48.76 | ⎯ | ⎯/3.53 | 11.07/2.82 | ⎯/8.38 | 11.07/11.2 | 18.67/3.88 | 1.26/6.71 | 44.30/25.44 |
| Japan [54] | 2005/2010 | 58.20/51 | ⎯ | ⎯/8.80 | 41.80/13.90 | 9.10/37.30 | 50.90/51.20 | 10.90/10.50 | 30/22.10 | 15.50/31.40 |
| India [55] | 2011-2013 | 100 | ⎯ | ≈4^j^ | ⎯ | ≈18 | ≈18 | ≈9 | ≈5 | ≈30 |
| Taiwan ^k^ [56] | 2000/2010 | ⎯ | ⎯ | ⎯ | ⎯ | ⎯ | ⎯ | ⎯ | ⎯ | ⎯ |
| South Africa [57] | 2010 | ⎯ | 46.50 | ⎯ | ⎯ | 39.80 | 39.80 | 2.12 | 1.80 | 9.20 |
| Sweden and Norway [84] | 2010-2013 | ⎯ | ≈59/76^l^ | ⎯ | ⎯ | ⎯ | ≈52/30 | ≈10/35^m^ | ⎯ | ⎯ |
| England ^n^[58] | 1999/2010 | ≈47.5/16 | ≈⎯/26 | ≈3.75/3.63 | ≈32.5/1.81 | ≈11.25/60.45 | ≈43.75/62.26 | ≈6.25/7.27 | ⎯ | ⎯ |
| USA/Japan [59] | 2003/2008^o^ | (70-80)/95 | ⎯ | 29/25 | ⎯ | ⎯ | 57/85 | ⎯/42 | ⎯ | ⎯ |
| Germany [60] | 2004 | ⎯ | 54.45/89.92^p^ | ⎯ | ⎯ | ⎯ | 79.58/43.41 | 14.13/13.17 | 23.56/24.03 | ⎯ |
| Japan ^q^[61] | 2005/2008 | 21/21 | ⎯ | ⎯/1 | 13/10 | 3.8/9 | 16.8/19 | 4/5 | 11/10 | 47.8/43 |
| Australia [62] | 1995/2009 | 36.50/52.30 | ⎯/55.90 | ⎯/2.90 | 4.10/4.80 | ⎯/5.90 | 4.10/10.90 | 7.90/2.10 | 2.90/3.50 | 48.70/24.10 |
| France ^r^ [63] | ≤2000/>2000 | ⎯ | ⎯ | ⎯ | ⎯ | ⎯ |  | ⎯ | ⎯ | ⎯ |
| USA[64] | 2000-2003 | ⎯ | 75.10 | 13.31 | ⎯ | ⎯ | 34.84 | 9.63 | 7.79 | 6.16 |
| Europe ^s^[65] | 2003/2007 | ⎯ | 25.63/30.49 | 10.50/5.61 | ⎯ | ⎯ | 53.69/54.06 | 3.89/5.80 | 1.86/1.10 | 4.40/2.91 |
| Italy [66] | 2003/2005^t^ | ≈49.91/43.73 | ⎯ | ⎯ | ≈8.48/13.53 | ≈16.63/18.51 | ≈25.11/32.04 | ⎯ | ⎯ | ≈24.95/24.21 |
| Spain [67] | 1992/2004 | 37.42/49.75 | ⎯ | ⎯/0.24 | 16.25/6.31 | ⎯/11.89 | 16.25/18.68 | 15.03/14.56 | ⎯ | 31.28/16.99 |
| USA [68] | 1998/2004 | 46.48/82.10^u^ | ⎯ | ⎯ | ⎯ | ⎯ | 19.81/7.63 | 0/1.67 | 14.41/4.29 | 18.91/3.81 |
| USA [69] | 1999-2001 | 70.92 | ⎯ | 1.01 | ⎯ | ⎯ | 13 | 5.05 | 5.92 | ⎯ |
| Singapore [70] | ⎯ | 92.3 | ⎯ | 6.8 | 26.8 | ⎯ | 26.8 | 21 | 2.9 | 22.9 |
| Spain [71] | 1999 | 90.4 | ⎯ | 5.8 | 28.9 | 15.1 | 44 | 31 | 2.8 | 9.6 |
| Sweden ^v^[72] | 1995/2001 | ≈50.54/63.84 | ⎯ | ≈⎯/7.50 | ⎯ | ⎯ | ≈1.44/9.21 | ≈48.1/19.79 | ⎯ | ⎯ |
| Italy [73] | 1997-1998 | 98.5 | ⎯ | 3.1 | 36.1 | 7.6 | 43.7 | 2.3 | 0.8 | 8.5 |
| USA [83] | 1992-1996 | 52.27 | ⎯ | ⎯ | ⎯ | ⎯ | 75 | 20.45 | ⎯ | 18.18 |
| Japan [74] | 1994-1996 | 78.84 | ⎯ | ⎯ | 76.92 | ⎯ | 76.92 | ⎯ | 44.23 | 30.76 |
| Italy [75] | 1986-1991 | 86.2 | ⎯ | ⎯ | ⎯ | ⎯ | ⎯ | 24.6 | ⎯ | ⎯ |

* A summary of the prescribing pattern or studies through which the percentage of patients prescribed each drug and drug class can be calculated (this includes studies that have examined the drug sales differences). Entries below the drug names are the percentages of all prescriptions, drug sales prices, or all patients for that row and year. If there are two years mentioned in the “year” column, they indicate the first and last year of the study if they are separated by slash symbol (/), and the prescription rates represented these years unless stated otherwise. If the two years are separated by the dash symbol (-), that means that the prescription rates were calculated for these years cross-sectionally at the same time unless stated otherwise. (Articles are ordered by year of publication).

1. “L-dopa only” = L-dopa + dopamine decarboxylase inhibitors (carbidopa or benserazide).
2. “L-dopa combination” = L-dopa + dopamine decarboxylase inhibitors and L-dopa + dopamine decarboxylase inhibitors + COMT inhibitors.
3. “—” = data unavailable or not applicable
4. L-dopa, levodopa; COMT, catechol-O-methyl transferases; DAs, dopamine agonists; MAO-B, monoamine oxidase inhibitors; NA, not applicable.
5. The study examined only L-dopa intestinal gel, so prescribing rate cannot be estimated.
6. Based on the data presented in the study, it was impossible to calculate prescription rates for every medication.
7. The study covered the period of 2007-2010, however, it did not examine the changes in the trend. Therefore, the last year of the study was included in the table, taken into account that there were no big differences in the prescription rates over the years of the study.
8. This study examined the difference in PD prescribing pattern between community and care homes. “/” = separates data for PD patients living in their homes and patients living in care homes, respectively.
9. This study examined the prescription rate of PD medications that used only by selegiline/rasagiline users. Therefore, the prescription rate for each PD medication/class cannot be calculated.
10. “≈” = the rate was estimated from a graph in the study and there were no specific numbers in the manuscript.
11. This study examined the pattern of initial therapy in PD patients from 2000 to 2010. It divided the years of the study to two periods: 2000-2005 and 2006-2010. Therefore, the total prescription rate per year cannot be calculated. The individual prescription rates of every PD medication cannot be calculated because the study has classified PD medications as L-dopa only (which means any L-dopa product with or without any PD medication other than DAs); and DAs only (which means any DA with or without any PD medication other than L-dopa).
12. “/” = separates data for PD patients in Sweden and Norway respectively.
13. The prescription rate of selegiline only.
14. This study examined the total net ingredient costs of PD medications in England between 1999 and 2010. All the sales percentage were estimated from a graph in the study because they were not mentioned in the manuscript.
15. This was a drug utilisation comparison study between the USA and Japan. The American study was conducted in 2003, and the Japanese study was conducted in 2008.
16. “/” = separates data for PD patients aged <70 years and PD patients aged >70 years in the year of study, respectively.
17. The study examined the effect of pergolide withdrawal from the USA market by applying a time interrupted series model. The year “2005” covered the period from September 2005–March 2007, while “2008” covered the period from April 2007–October 2008.
18. The study did not specify exactly in which year the prescription rates were calculated. Additionally, all medications were presented as a combination with other medications, therefore, the prescription rates cannot be calculated.
19. The study examined the changes in PD medications sales in 26 European countries. The unit of analysis was DID (DDD per 1000 inhabitants daily). DID cannot be calculated for the whole of Europe. However, the difference in PD medication sale prices between 2003 and 2007 was calculated and presented in the table.
20. “/” = separates data for the difference in percentage of the prevalence of a particular PD medication use per 100,000 inhabitants out of the total percentages of prevalence of all PD medications use per 100,000 inhabitants between 2003 and 2005.
21. The study examined the initial antiparkinsonian therapy in newly diagnosed PD patients from 1998 to 2004. “/” = separates data for PD patients aged <65 years and PD patients aged ≥65 years in the years of the study, respectively.
22. The study examined the changes in PD medication sales in Sweden. The unit of analysis was DID (DDD per 1000 inhabitants daily). The difference in PD medications DIDs between 1995 and 2001 was estimated from a graph in the study and presented in the table.
